# Assessment of the Nutritional Impact of the 10% Snack Recommendation in Pet Diets

**DOI:** 10.3390/vetsci12030282

**Published:** 2025-03-18

**Authors:** Leonardo de Andrade Príncipe, Pedro Henrique Marchi, Andressa Rodrigues Amaral, Vivian Pedrinelli, Rafael Vessecchi Amorim Zafalon, Felipe Saab Romano, Júlio Cesar de Carvalho Balieiro, Thiago Henrique Annibale Vendramini

**Affiliations:** 1 Pet Nutrology Research Center (CEPEN Pet), Department of Animal Nutrition and Production, School of Veterinary Medicine and Animal Science, University of Sao Paulo, Pirassununga 13635-000, Brazil; leoprincipe@usp.br (L.d.A.P.); pedro.henrique.marchi@usp.br (P.H.M.); rafael.zafalon@usp.br (R.V.A.Z.); balieiro@usp.br (J.C.d.C.B.); 2 Veterinary Nutrology Service, Teaching Veterinary Hospital, School of Veterinary Medicine and Animal Science, University of Sao Paulo, Sao Paulo 05508-270, Brazilvivian.pedrinelli@gmail.com (V.P.); 3 Ferogastro, Brazilian Association of Animal Gastroenterology (ABRAGA), Sao Paulo 04062-003, Brazil; felipe.med.vet@hotmail.com

**Keywords:** canine, fat imbalance, feeding practices, feline, macronutrients, protein imbalance

## Abstract

The World Small Animal Veterinary Association (WSAVA) and the American Animal Hospital Association (AAHA) recommend that treats make up no more than 10% of daily energy intake for dogs and cats. This study assessed whether replacing 10% of maintenance energy requirements with commercial treats affects the nutritional adequacy of diets. Labels from 226 dry extruded diets for adult dogs and 124 for adult cats in the Brazilian market were analyzed, along with 170 dog treats and 114 cat treats (dry, wet, or liquid). Maintenance energy requirement calculations followed FEDIAF (2024) guidelines. All diets for active and inactive dogs met protein and fat requirements after a 10% intake reduction and the inclusion of any type of snacks. Regarding protein intake, all diets met the minimum requirement for active cats. However, for inactive cats, some diets failed to provide enough crude protein after restriction (36.29% of diets with dry snacks, 16.12% with wet snacks, and 2.41% with liquid snacks). Concerning fat content, only 1.61% of diets failed to meet the minimum when liquid snacks were included. On the other hand, when considering the equation and the needs of inactive cats, 29.03% of diets with dry snacks, 28.22% with wet snacks, and 44.77% with liquid snacks did not meet the minimum. Reducing food intake to accommodate treats may not be ideal, depending on diet composition and treat selection, particularly for neutered and indoor cats.

## Simple Summary

It is essential for pet owners to understand how the food they provide impacts their pets’ health. This study examined whether replacing 10% of the daily energy needs with treats affects the nutritional quality of diets. The research analyzed the nutritional information labels of various commercial dry foods and commercial snacks for both healthy adult dogs and cats. We analyzed 226 dog food labels and 124 cat food labels in the Brazilian market, as well as 170 dog treats and 114 cat treats. Our results showed that all diets for active and inactive dogs met protein and fat requirements, even with the inclusion of treats. For active cats, all diets met the minimum protein requirement, but for inactive cats, some diets with dry, wet, or liquid treats did not meet the protein and fat requirements. Reducing food intake to fit treats may not be ideal for some cats, especially neutered or indoor cats, as it could lead to nutrient deficiencies.

## 1. Introduction

The practice of providing snacks and treats is widespread among dog and cat owners, integrating these items into their pets’ diets in various ways, which form a significant component of their dietary regimens [1,2]. These snacks and treats are often used for a variety of purposes, such as training rewards, positive reinforcement, enrichment, alleviating owner guilt, responding to marketing pressures, serving as food toppers, or even as a form of supplemental feeding. However, the excessive consumption of treats, when not properly balanced with the rest of the diet, can contribute to obesity [3], while replacing complete and balanced meals with snacks may lead to deficiencies in essential nutrients.

However, most owners do not consider snacks and treats as essential components of their pets’ nutritional intake. As a result, the choices made by pet owners are often influenced more by non-nutritional attributes such as flavor, shape, and color, rather than by nutritional value. This can lead to an imbalance, where snacks contribute to excessive caloric intake, exacerbating the risk of obesity, or replace essential nutrients in the diet, potentially leading to nutritional deficiencies. These practices, often driven by marketing strategies, overlook the importance of a well-rounded diet, placing pets at risk for both obesity and malnutrition [1,4,5].

The World Small Animal Veterinary Association (WSAVA) and the American Animal Hospital Association (AAHA) recommend that up to 10% of the daily caloric intake for dogs and cats can come from non-complete foods [6,7,8]. This recommendation aims to balance the inclusion of snacks and treats, helping to prevent obesity. However, while the 10% guideline is designed to mitigate the risk of excess caloric intake leading to obesity, it is also essential to ensure that the remaining 90% of the diet continues to provide complete and balanced nutrition. If too much of the diet is replaced by snacks, even within the 10% limit, there may be a risk of nutritional deficiencies, particularly of essential nutrients such as proteins and fats.

The nutritional and dietary recommendations proposed by Chandler and Takashima [9] emphasize that a complete and balanced base diet is the main source of essential nutrients. However, the authors recognize the importance of considering the inclusion of additional treats and supplements, since they are part of the dietary management of dogs. They emphasize the role of veterinarians in educating owners about the caloric contribution of these foods and the need for control to preserve the health of their animals [9]. The need to educate owners and account for the number of treats provided arises from the fact that many owners often ignore additional sources of calories in their pets’ diets. To facilitate this control, Chandler and Takashima suggest that treats should not exceed 10% of the total daily caloric intake, ensuring that the diet remains complete and balanced. However, this recommendation assumes that the remaining 90% of the diet meets all nutritional requirements [9].

With the increasing adoption of unconventional diets, maintaining an adequate balance of nutrients becomes more challenging. The transition to these alternative feeding practices raises concerns about nutritional adequacy, as owners may inadvertently exceed safe limits for supplemental foods, thereby increasing the risk of imbalanced nutrition [8,10]. Management errors also play a significant role, with the widespread availability of snacks and treats being a key concern [11,12,13]. Although providing dry extruded commercial foods in the correct amounts is not inherently associated with obesity [5], there remains a substantial gap in pet caregivers’ awareness and understanding of proper portioning of both food and treats. However, the risks associated with improper snack and treat consumption are not limited to obesity. Excessive intake of snacks, especially when used to replace complete and balanced meals, can not only contribute to obesity but also result in deficiencies in essential nutrients, such as protein and fat, that are crucial for overall health.

Studies have evaluated the perceptions, practices, and behaviors of owners regarding food management for their pets [1,5,10,12,14]. However, none have assessed the impact of these practices on meeting nutritional needs, particularly regarding essential nutrients such as protein and fat. The growing tendency to offer snacks and treats, often chosen for taste rather than nutritional value, may contribute to deficiencies. This highlights the need to assess whether current recommendations ensure nutritional adequacy. Therefore, this study aimed to evaluate whether the WSAVA and AAHA guidelines, which allow up to 10% of the maintenance energy requirement (MER) to come from snacks and treats, represent a safe practice for dogs and cats, considering potential nutrient deficiencies.

## 2. Materials and Methods

A total of 226 labels from dry extruded complete diets for healthy adult dogs and 124 labels from dry extruded complete diets for healthy adult cats were evaluated. These diets are all commercially available from leading brands of pet food in the Brazilian market, specifically for adult dogs and cats. Diets for puppies, senior dogs and cats, and clinical nutrition diets for pets with specific clinical conditions were not included. The protein and fat contents were analyzed based on the information declared on the labels. The average protein and fat contents for each category were calculated to determine a standard snack profile for inclusion calculations. The MER was calculated as follows: 95 kcal × BW^0.75^ for inactive dogs, 75 kcal × BW^0.67^ for inactive cats, 110 kcal × BW^0.75^ for active dogs, and 100 kcal × BW^0.67^ for active cats, in accordance with European Pet Food Industry Federation (FEDIAF) [15].

The MER included a 10% contribution from dietary additions, following WSAVA and AAHA guidelines, which consider all types of treats. A random sample of major commercial dog (*n* = 170) and cat (*n* = 114) snack labels was selected based on the most widely available products in the Brazilian market. Additionally, their nutritional composition was assessed according to the protein and fat contents declared on the labels. All products were classified into dry (dog = 155; cat = 61), wet (dog = 27; cat = 15), and liquid (cat = 26) categories based on the moisture content stated on the label. Readability could also be improved by stating that 90% of the intended dietary intake was calculated from on-pack label information to evaluate the recommended 10% limit on replacement of the main meal. Consequently, only 10% of total food intake was subtracted, focusing the nutritional assessment on the remaining portion of the diet.

## 3. Results

**Figure 1:**
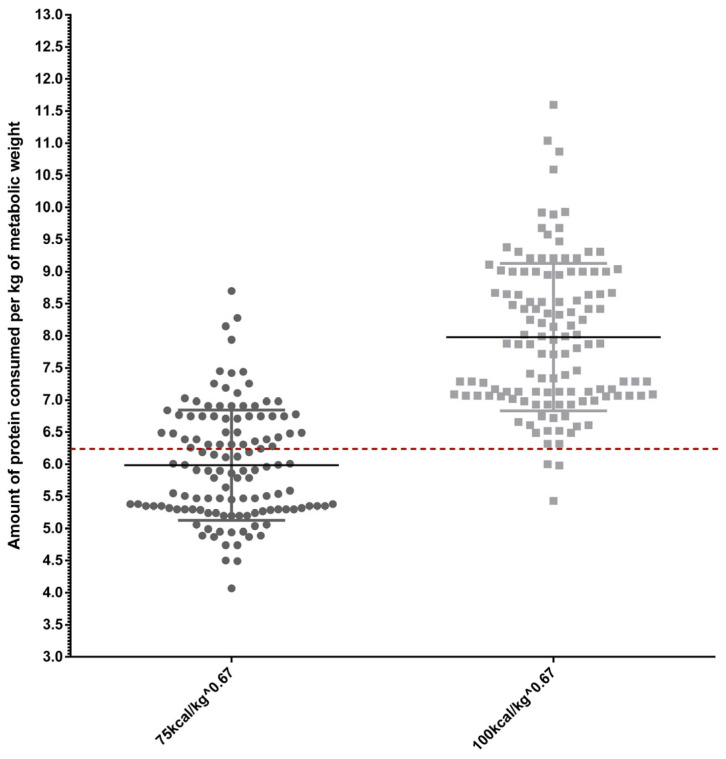
Distribution of protein intake in cat diets (g/kg of metabolic body weight) following a 10% restriction of the maintenance energy requirement. The dashed line represents the FEDIAF [15] minimum requirement for protein intake.

**Figure 2:**
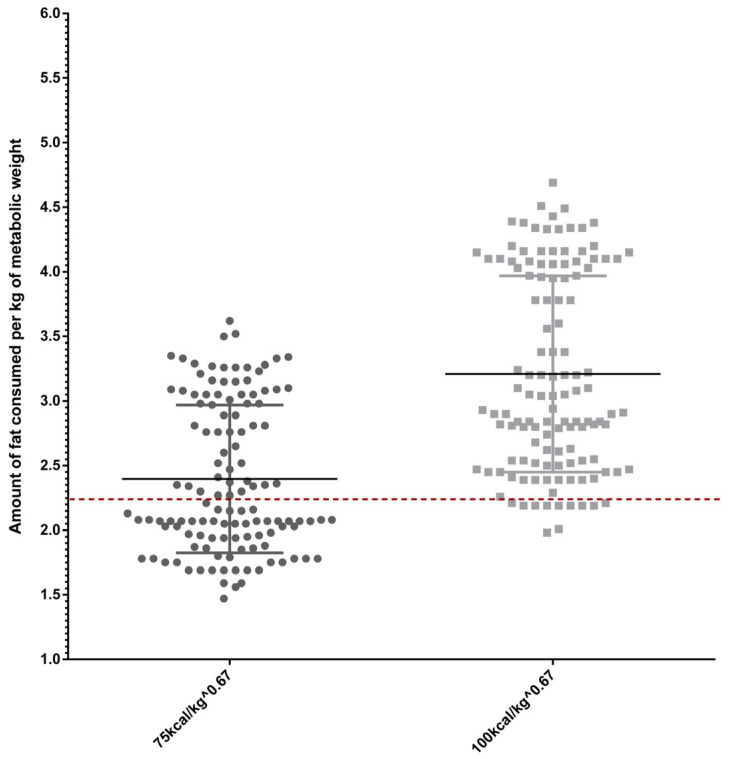
Distribution of fat intake in cat diets (g/kg of metabolic body weight) following a 10% restriction of the maintenance energy requirement. The dashed line represents the FEDIAF [15] minimum requirement for protein intake.

According to FEDIAF [15] minimum protein and fat recommendations, all evaluated diets provided these nutrients at adequate levels, even before replacing 10% of the maintenance energy requirement per kilogram of metabolic body weight for both inactive and active dogs with the average protein and fat values of snacks. For active cats, most diets met protein and fat requirements when calculated per kilogram of metabolic weight under a 10% intake reduction without treat inclusion, although some diets were found to lack adequate protein and fat content. In contrast, more than half of the diets for inactive cats were insufficient to meet the nutritional demands for protein and fat under restricted intake conditions without treat inclusion. Figure 1 and Figure 2 provide graphical representations of protein and fat intake per kilogram of metabolic weight in cats, aligned with FEDIAF (2024) recommendations.

The analysis results, considering a 10% reduction based on the equation for active and inactive dogs and cats, along with the inclusion of 10% of the main commercial snacks available on the market, are presented in Supplementary Tables S1 and S2 for protein intake in dogs and cats, respectively, and in Supplementary Tables S3 and S4 for fat intake in dogs and cats, respectively. Regarding the protein and fat content reported on dog food labels, all analyzed diets met or exceeded the minimum recommended levels for both active and inactive dogs.

Similarly, all diets for active cats provided sufficient protein to meet the minimum requirements set by FEDIAF. For inactive cats, 36.29% (*n* = 45/124) of diets with dry snacks, 16.12% (*n* = 20/124) of diets with wet snacks, and 2.41% (*n* = 3/124) of diets with liquid snacks did not meet FEDIAF’s minimum protein requirements. Regarding fat content, 1.61% (*n* = 2/124) of diets with liquid snacks for active cats failed to meet the minimum recommended fat levels. In contrast, for inactive cats, 29.03% (*n* = 36/124) of diets with dry snacks, 28.22% (*n* = 35/124) of diets with wet snacks, and 44.77% (*n* = 58/124) of diets with liquid snacks did not meet FEDIAF’s minimum fat requirements (Table 1).

## 4. Discussion

Several studies have reported the progressive increase in the pet food practice of offering snacks to dogs and cats [2,10,16,17]. Morelli et al. (2020) observed that there were several justifications for this practice, including the need for positive reinforcement during sport conditioning and training [1]. Additionally, respondents also cited motives such as fortifying the bond between humans and their pets, creating enjoyable moments within the daily routine, and expressing affection [1]. Moreover, socioeconomic investigations have revealed that, in comparison to other products within the pet industry, dog and cat owners allocate a substantial portion of their expenditures towards food and snacks [18,19,20,21].

According to Brazilian regulations, treats intended for dogs and cats are classified as specific foods. These products are not designed to be complete and balanced, as their primary purpose is recreational and emotional, fostering a bond between humans and their pets [22]. Consequently, these treats are often perceived as having minimal nutritional value, with a negligible impact on the overall nutrient composition of the diet [4]. However, in Brazil, there is no legislation mandating the inclusion of minimum or maximum nutrient levels for pet food products. While it is customary for Brazilian brands to declare minimum nutrient levels, as is common in other countries, studies have frequently highlighted inconsistencies between the nutritional information stated on labels and the results of laboratory analyses of dog and cat foods [23,24,25].

In some cases, variance between declared and actual nutrient levels reveals values significantly exceeding the minimum, which can pose a risk of overfeeding certain nutrients and contribute to health issues such as obesity. These differences may also vary depending on the regulatory practices of different countries, as some regions require additional information, such as maximum nutrient levels, while others focus primarily on minimum levels. This lack of uniformity in labeling practices could lead to challenges in interpreting the information provided on pet food labels [23,24,25].

The wide variety of snack types available could further exacerbate the potential for protein and/or fat imbalances when the 10% dietary restriction is applied for their inclusion. Addressing these issues requires greater attention to labeling practices, ensuring consumers have accurate and consistent information to guide feeding decisions. Additionally, this highlights the importance of initiating a broader discussion about the risks of generalizing dietary recommendations. Evaluating the specific diet that the patient is consuming becomes essential for tailoring appropriate nutritional strategies and mitigating potential imbalances.

The nutritional guidelines for dogs and cats, as outlined by FEDIAF (2024) [15] and NRC (2006) [26], include minimum levels of protein and fat, in addition to amino acids and essential fatty acids. The findings of this study suggest that a significant proportion of cat diets exhibited an imbalance in protein and fat content under a 10% caloric restriction. According to the NRC (2006) [26], the minimal requirement of crude protein and total fat for adult dogs for maintenance is 2.62 g and 1.8 g per kg of metabolic bodyweight, respectively. For adult cats, the NRC recommended a minimal requirement of 3.97 g per kg of metabolic bodyweight for crude protein and 2.2 g per kg of metabolic bodyweight of total fat. Furthermore, the energy requirements for adult dogs and cats at maintenance are different in NRC.

For dogs, the metabolizable energy requirements are 130 kcal × BW^0.75^ and 95 kcal × BW^0.75^ for active and inactive dogs, respectively. According to the FEDIAF guidelines, the energy requirements for active dogs vary depending on the level of activity: 95 kcal × BW^0.75^ for low activity (e.g., walking on a lead for less than 1 h/day), 110 kcal × BW^0.75^ for moderate activity (1–3 h/day of low-impact activity), 125 kcal × BW^0.75^ for moderate activity with high impact (1–3 h/day), and between 150 and 175 kcal × BW^0.75^ for high activity (3–6 h/day, such as working dogs like sheepdogs). In extreme conditions, such as for racing sled dogs (168 km/day in extreme cold), the requirement can range from 860 to 1240 kcal × BW^0.75^. The same change is observed for daily metabolizable energy requirements for adult cats at maintenance is 130 kcal × BW^0.4^ for overweight and obese cats (inactive) and 100 kcal × BW^0.67^ for lean cats (active) (FEDIAF castrated or inactive: 75 kcal × BW^0.67^) [15].

The differences in literature between NRC and FEDIAF are very important. Comparing all the cats and dogs’ diets analyzed in the study and considering the NRC’s minimal requirements for crude protein and total fat with the predictive equation’s recommendations for dogs and cats, we observed that all the diets for dogs and cats, according to the level of activity, achieved the minimal protein and fat requirement after 10% MER intake restriction and the inclusion of all snack types. No possible nutritional deficiencies were observed in the nutritional management of the inclusion of snacks, according to the WSAVA, based on the NRC. This happens because the minimal nutrient requirements are less in NRC, and the equations result in a larger calorie intake than in FEDIAF. This raises an important discussion regarding the choice of nutritional references veterinarians should rely on when formulating diet plans for their patients. While the FEDIAF nutritional guidelines are updated annually and are more accessible, reflecting the latest scientific knowledge and advancements in pet nutrition, the NRC guidelines, although historically significant, may be more applicable to certain commercially available diets.

The predictive equations proposed by the NRC may not fully reflect the current lifestyle and energy demands of dogs and cats, often leading to an overestimation of their caloric needs. Modern pet ownership is characterized by emotional bonds, with feeding practices playing one of the central roles in the human–animal relationship [2]. This is particularly evident in the growing trend of treat provisioning, as many owners perceive offering food as an expression of affection [27]. For example, some pet caregivers prefer unprocessed treats, believing them to be a more natural and healthier choice for their dogs [28]. The differences between the guidelines and the new perspectives on the human-animal bond highlight the need for veterinarians to consider nutritional demands as unique to each patient, especially in relation to current nutritional guidelines, which are sometimes generalist, and the specific diet and treat formulations available on the market.

Similarly, the WSAVA is a global organization that provides guidelines and recommendations for veterinarians worldwide. However, due to differences between the FEDIAF and NRC guidelines, certain aspects of the global guidelines may be incomplete or inconsistent. For example, the recommendation that snacks should constitute 10% of the daily MER for dogs and cats may not be universally applicable across all guidelines, as there are variations in how these organizations approach nutritional requirements. This study highlights the need for more careful consideration when applying such recommendations to cats, given their distinct nutritional needs, which differ significantly from those of dogs [15,26]. As such, it is crucial to develop guidelines that are specifically tailored to the unique needs of each species to ensure optimal health and nutrition.

As demonstrated by the findings of this study, when applying the predictive equations for the metabolizable energy requirements established by FEDIAF [15], together with the minimum protein and fat requirements, certain considerations should be made regarding the inclusion of treats in the dietary management of inactive cats. After implementing a 10% reduction in MER and incorporating commercial treats, the minimum protein and fat requirements established by FEDIAF [15] were not met. In addition, it was observed that depending on the presentation of the treat (dry, wet, or liquid), the percentage of dry diets that met the minimum protein and fat requirements varied for cats.

Cats exhibit a higher dietary requirement for protein and fat due to their unique metabolic characteristics. Inadequate levels of these macronutrients, particularly when accompanied by imbalances or deficiencies in essential amino acids or fatty acids, may pose significant health risks [26]. Additionally, it is worth noting that cats are glyconeogenic animals, meaning they utilize the carbon chain of certain amino acids to synthesize glucose and generate energy [29]. Consequently, the provision of a protein-rich diet is of greater importance for cats compared to dogs, as it is crucial for preserving lean muscle mass in this species. Furthermore, the restriction of essential amino acids in cat food can result in hyperammonemia and uremia [30], decreased growth rate [31], changes in nitrogen metabolism, dermatitis [32], and reproductive [33] and cardiac dysfunctions [34]. For example, arginine and taurine are essential amino acids for cats and must be present in diets in adequate amounts [26].

Cats also had a limited capacity to synthesize arginine due to the low activity of pyrroline-5-carboxylate (P5C) synthase [35]. Morris and Rogers (1978) [32] observed that cats that consumed an arginine-deficient diet presented hyporexia, hyperammonemia, neurological signs, severe emesis, ataxia, tetanic spasms, and death. Moreover, cats have a restricted ability to synthesize taurine because of the low action of the enzyme’s cysteine dioxygenase and cysteine sulfinic acid decarboxylase in the conversion of cystine into taurine [36]. Deficiency in taurine supplementation can result in reproductive problems, blindness, deafness, central retinal degeneration, dilated cardiomyopathy, and heart failure [35]. A reduced fat intake can lead to a deficiency in essential fatty acids, notably arachidonic acid. Arachidonic acid is a crucial component of the feline diet, as cats possess limited capacity to convert linoleic acid into arachidonic acid, primarily due to the low enzymatic activity of alpha-6 desaturase [37]. Arachidonic acid deficiency may be related to hematological manifestations of thrombocytopenia and platelet aggregation, as well as reproductive disorders in cats [38].

This concern becomes even more relevant when we consider the unique nutritional requirements of cats, especially their dependence on protein and fat. Although global organizations support the inclusion of treats based on MER, this approach may inadvertently reduce intake of essential nutrients, potentially leading to amino acid and fatty acid deficiencies. Given the substantial presence of commercial pet foods, our findings suggest that after caloric restriction, some of these diets may not fully meet the minimum standards recommended by FEDIAF [15], even when commercial treats are included. It is important to acknowledge the potential for nutritional deficiencies, including other essential nutrients, following a 10% caloric restriction. While our study focused on protein and fat intake, the lack of calculations for other essential nutrients, such as vitamins and minerals, represents an important limitation. If even these key macronutrients may fail to meet the recommended minimum levels after caloric restriction and addition of treats, it is minimally reasonable to consider that micronutrient deficiencies could also arise, given their lower dietary concentrations and higher variability among dry diets and snack formulations.

Furthermore, veterinarians also face the challenge of inconsistencies in the accuracy of information provided on dog and cat food labels. As noted by Zafalon et al. (2020) [39], discrepancies were observed between the bromatological analyses of specific nutrients and their respective values as indicated on the labels. The authors found that 48.55% of the products exhibited a lower fat content than what was stated on the label. Notably, no prior studies have undertaken an analysis of the protein content in dog and cat treats. Olivry and Mueller (2018) [26] suggest that the amount of incorrectly labelled commercial dog and cat foods is common, and while the exact frequency is not precisely known, it is likely to surpass what is currently reported in the literature. Discrepancies arise between the analyses conducted by manufacturers for label preparation and those performed in independent laboratories. Hill et al. (2009) [25] observed divergences, for lower values, of up to 1.5% and 1.0% of protein and fat on commercial food labels. Conversely, some discrepancies also reveal higher values than those reported on the label, which warrants additional consideration of nutrient excess, a critical aspect that should also be addressed when initiating this discussion. The discrepancy between the stated levels and actual nutrient content of foods heightens the concern surrounding this deficiency and excess scenario.

Pet food companies are encouraged to provide detailed labeling information, including the recommended amounts of food for bodyweight ranges and activity levels while accounting for the inclusion of treats, based on their specific formulations. This approach would reflect the current feeding practices of family dogs and cats, where a combination of complete foods and treats is prevalent. By moving beyond the generic 10% treat guideline, this initiative could establish a more accurate and globally applicable recommendation, benefiting both pet caregivers and the veterinary community worldwide. The authors acknowledge the methodological limitations inherent in food label analysis, including the lack of detailed information on fat types, amino acid composition of proteins, digestibility, bioavailability, and biological value of nutrients, as well as the reliance on self-reported data that may not accurately reflect the actual nutrient content of products and the lack of direct assessments of specific nutrients.

The methodological approach of this study can be considered investigative, with the aim of highlighting the relevance of this issue rather than providing definitive conclusions. The intention of our work is not to diagnose deficiencies or make conclusive claims, but to raise critical questions about the adequacy of current nutritional guidelines and their alignment with the realities of the pet food market.

## 5. Conclusions

Reducing the amount of food provided in the daily dietary intake of cats and dogs to include treats may imply a reduced intake of essential nutrients. Despite the guidance of WSAVA and AAHA, the practice of allocating up to 10% of the total caloric intake for treats may warrant reconsideration, particularly for neutered and/or indoor cats that consume less than manufacturer recommendations.

## Figures and Tables

**Table 1:** Nutritional adequacy of protein and fat in commercial dog and cat foods: an analysis based on activity levels and compliance with recommendations according FEDIAF (2024) and inclusion of snacks according WSAVA and AAHA.

Protein					
Category	Number of Diets Meeting Requirements at 10% Reduction Alone	Number of Diets Meeting Requirements with Average Snack Nutrition Accounted	Number of Diets Not Meeting Requirements Even Accounting for Snack Nutrition	Diets Meeting or Exceeding Minimum with Snacks (%)	Diets Below Minimum with Snacks Inclusion (%)
Active dog (110 × BW^0.75^)	224	-	-	100.0%	-
Inactive dog (95 × BW^0.75^)	218	6	-	100.0%	-
Active cat (100 × BW^0.67^)	121	3	-	100.0%	-
Inactive cat (75 × BW^0.67^)	49	75	45	With dry snacks: 63.71% (*n* = 79/124) With wet snacks: 83.88% (*n* = 104/124) With liquid snacks: 97.59% (*n* = 121/124)	With dry snacks: 36.29% (*n* = 45/124) With wet snacks: 16.12% (*n* = 20/124) With liquid snacks: 2.41% (*n* = 3/124)
**Fat**					
**Category**	**Number of Diets** **Meeting Requirements** **at 10% Reduction Alone**	**Number of Diets** **Meeting Requirements** **with Average Snack** **Nutrition Accounted**	**Number of Diets** **Not Meeting** **Requirements Even** **Accounting for** **Snack Nutrition**	**Diets Meeting or** **Exceeding** **Minimum** **with Snacks (%)**	**Diets Below** **Minimum** **with Snacks** **Inclusion (%)**
Active dog (110 × BW^0.75^)	224	-	-	100.0%	-
Inactive dog (95 × BW^0.75^)	224	-	-	100.0%	-
Active cat (100 × BW^0.67^)	113	11	2	With liquid snacks: 98.39% (*n* = 122/124)	With liquid snacks: 1.61% (*n* = 2/124)
Inactive cat (75 × BW^0.67^)	60	64	58	With dry snacks: 70.97% (*n* = 88/124) With wet snacks: 71.78% (*n* = 89/124) With liquid snacks: 55.23% (*n* = 66/124)	With dry snacks: 29.03% (*n* = 36/124) With wet snacks: 28.22% (*n* = 35/124) With liquid snacks: 44.77% (*n* = 58/124)
Legend: BW = body weight					

## Data Availability

The data analyzed in this investigation are available upon request to the corresponding author.

## Supplementary Materials

The following supporting information can be downloaded at: https://www.mdpi.com/article/10.3390/vetsci12030282/s1. Table S1: Nutritional impact of 10% MER restriction with snack inclusion in protein intake of dogs, according to FEDIAF (2024). Table S2: Nutritional impact of 10% MER restriction with snack inclusion in protein intake of cats, according to FEDIAF (2024). Table S3: Nutritional impact of 10% MER restriction with snack inclusion in fat intake of dogs, according to FEDIAF (2024). Table S4: Nutritional impact of 10% MER restriction with snack inclusion in fat intake of cats, according to FEDIAF (2024).
