# Supplementary material for: Assessment of the Nutritional Impact of the 10% Snack Recommendation in Pet Diets
Source: Vet Sci. 2025 Mar 18;12(3):282. doi: 10.3390/vetsci12030282 (PMC11945345; doi:10.3390/vetsci12030282)
Supplement: Supplementary file 1 [file vetsci-12-00282-s001.zip › Supplementary_Table1_DogProtein.pdf]

Supplementary Table 1. Nutritional impact of 10% MER restriction with snack inclusion in protein intake of dogs. according FEDIAF (2024)

					Protein							
					95 kcal/kg$^{0.75}$				110 kcal/kg$^{0.75}$			
					Minimum recommended protein 4.95 g per kg metabolic bodyweight							
Commercial dry diet	Brand	Metabolizable energy (kcal/kg)	Crude protein (g/kg)	Fat (g/kg)	Food consumption considering 90% of the maintenance energy requirement. per kg of metabolic weight	Amount of protein consumed per kg of metabolic weight	Protein consumed per kg of (BW)0.75 + protein from dry snacks	Protein consumed per kg of (BW)0.75 + protein from wet snacks	Food consumption considering 90% of the maintenance energy requirement. per kg of metabolic weight	Amount of protein consumed per kg of metabolic weight	Protein consumed per kg of (BW)0.75 + protein from dry snacks	Protein consumed per kg of (BW)0.75 + protein from wet snacks
1	A	3869	280	160	22.10	6.19	6.94	7.43	25.59	7.16	8.04	8.61
2	A	4058	280	180	21.07	5.90	6.66	7.14	24.40	6.83	7.71	8.27
3	A	3901	280	140	21.92	6.14	6.89	7.38	25.38	7.11	7.98	8.55
4	A	3667	270	140	23.32	6.30	7.05	7.54	27.00	7.29	8.17	8.73
5	A	3888	270	160	21.99	5.94	6.69	7.18	25.46	6.88	7.75	8.32
6	A	3783	290	140	22.60	6.55	7.31	7.80	26.17	7.59	8.47	9.03
7	A	3899	260	160	21.93	5.70	6.46	6.95	25.39	6.60	7.48	8.04
8	A	3993	270	180	21.41	5.78	6.54	7.03	24.79	6.69	7.57	8.14
9	A	4124	290	180	20.73	6.01	6.77	7.26	24.01	6.96	7.84	8.40
10	A	4142	290	180	20.64	5.99	6.74	7.23	23.90	6.93	7.81	8.37
11	A	4142	300	180	20.64	6.19	6.95	7.44	23.90	7.17	8.05	8.61
12	A	3598	230	110	23.76	5.47	6.22	6.71	27.52	6.33	7.21	7.77
13	A	3667	270	140	23.32	6.30	7.05	7.54	27.00	7.29	8.17	8.73
14	A	3695	280	110	23.14	6.48	7.24	7.72	26.79	7.50	8.38	8.94
15	A	3830	310	120	22.32	6.92	7.68	8.16	25.85	8.01	8.89	9.45
16	A	3961	220	180	21.59	4.75	5.51	5.99	24.99	5.50	6.38	6.94
17	A	3964	260	160	21.57	5.61	6.37	6.85	24.97	6.49	7.37	7.93
18	A	4095	240	180	20.88	5.01	5.77	6.26	24.18	5.80	6.68	7.24
19	A	3691	220	120	23.16	5.10	5.85	6.34	26.82	5.90	6.78	7.34
20	A	3859	260	120	22.16	5.76	6.52	7.01	25.65	6.67	7.55	8.11
21	A	4092	240	160	20.89	5.01	5.77	6.26	24.19	5.81	6.68	7.25
22	A	3953	220	170	21.63	4.76	5.52	6.00	25.04	5.51	6.39	6.95
23	A	4156	220	160	20.57	4.53	5.28	5.77	23.82	5.24	6.12	6.68
24	A	4055	240	180	21.09	5.06	5.82	6.31	24.41	5.86	6.74	7.30
25	A	4003	240	150	21.36	5.13	5.88	6.37	24.73	5.94	6.81	7.38
26	A	3920	220	150	21.81	4.80	5.56	6.04	25.26	5.56	6.43	7.00
27	A	4009	250	140	21.33	5.33	6.09	6.58	24.69	6.17	7.05	7.61
28	A	3839	230	120	22.27	5.12	5.88	6.37	25.79	5.93	6.81	7.37
29	A	3963	240	150	21.57	5.18	5.93	6.42	24.98	6.00	6.87	7.44
30	A	4161	260	180	20.55	5.34	6.10	6.59	23.79	6.19	7.06	7.63
31	A	3118	320	80	27.42	8.77	9.53	10.02	31.75	10.16	11.04	11.60
32	A	3144	250	90	27.19	6.80	7.56	8.04	31.49	7.87	8.75	9.31
33	A	3263	280	90	26.20	7.34	8.09	8.58	30.34	8.50	9.37	9.94
34	A	3165	250	90	27.01	6.75	7.51	8.00	31.28	7.82	8.70	9.26
35	B	3800	280	130	22.50	6.30	7.06	7.54	26.05	7.29	8.17	8.74
36	B	3980	260	150	21.48	5.59	6.34	6.83	24.87	6.47	7.34	7.91
37	B	3740	280	120	22.86	6.40	7.16	7.65	26.47	7.41	8.29	8.85
38	B	3810	240	105	22.44	5.39	6.14	6.63	25.98	6.24	7.11	7.68

39	C	4097	290	180	20.87	6.05	6.81	7.30	24.16	7.01	7.88	8.45
40	C	4123	290	180	20.74	6.01	6.77	7.26	24.01	6.96	7.84	8.40
41	C	3931	290	140	21.75	6.31	7.06	7.55	25.18	7.30	8.18	8.74
42	C	4213	290	190	20.29	5.89	6.64	7.13	23.50	6.81	7.69	8.26
43	C	4020	280	160	21.27	5.96	6.71	7.20	24.63	6.90	7.77	8.34
44	C	4040	260	160	21.16	5.50	6.26	6.75	24.50	6.37	7.25	7.81
45	C	4040	260	160	21.16	5.50	6.26	6.75	24.50	6.37	7.25	7.81
46	C	3920	260	140	21.81	5.67	6.43	6.92	25.26	6.57	7.44	8.01
47	C	3555	280	80	24.05	6.73	7.49	7.98	27.85	7.80	8.67	9.24
48	C	3555	280	80	24.05	6.73	7.49	7.98	27.85	7.80	8.67	9.24
49	C	3601	315	115	23.74	7.48	8.24	8.72	27.49	8.66	9.54	10.10
50	C	4015	250	160	21.30	5.32	6.08	6.57	24.66	6.16	7.04	7.61
51	C	3724	260	140	22.96	5.97	6.73	7.21	26.58	6.91	7.79	8.35
52	C	2979	355	80	28.70	10.19	10.95	11.43	33.23	11.80	12.67	13.24
53	C	2979	355	80	28.70	10.19	10.95	11.43	33.23	11.80	12.67	13.24
54	C	3550	340	140	24.08	8.19	8.95	9.43	27.89	9.48	10.36	10.92
55	C	3550	340	140	24.08	8.19	8.95	9.43	27.89	9.48	10.36	10.92
56	C	3831	200	130	22.32	4.46	5.22	5.71	25.84	5.17	6.04	6.61
57	C	4000	240	180	21.38	5.13	5.89	6.37	24.75	5.94	6.82	7.38
58	D	3875	311	211	22.06	6.86	7.62	8.11	25.55	7.95	8.82	9.39
59	D	4250	300	180	20.12	6.04	6.79	7.28	23.29	6.99	7.86	8.43
60	D	4250	300	180	20.12	6.04	6.79	7.28	23.29	6.99	7.86	8.43
61	D	4150	280	170	20.60	5.77	6.53	7.01	23.86	6.68	7.56	8.12
62	D	4150	280	170	20.60	5.77	6.53	7.01	23.86	6.68	7.56	8.12
63	D	4150	280	170	20.60	5.77	6.53	7.01	23.86	6.68	7.56	8.12
64	D	4050	280	160	21.11	5.91	6.67	7.16	24.44	6.84	7.72	8.29
65	D	4050	280	160	21.11	5.91	6.67	7.16	24.44	6.84	7.72	8.29
66	D	4050	280	160	21.11	5.91	6.67	7.16	24.44	6.84	7.72	8.29
67	D	3610	264	165	23.68	6.25	7.00	7.49	27.42	7.23	8.11	8.67
68	D	3480	286	132	24.57	7.02	7.78	8.26	28.45	8.13	9.00	9.57
69	D	3680	275	165	23.23	6.38	7.14	7.63	26.90	7.39	8.27	8.83
70	D	3870	308	187	22.09	6.80	7.56	8.04	25.58	7.87	8.75	9.31
71	E	3660	240	120	23.36	5.61	6.36	6.85	27.05	6.49	7.37	7.93
72	E	3660	240	120	23.36	5.61	6.36	6.85	27.05	6.49	7.37	7.93
73	E	3840	330	198	22.27	7.34	8.10	8.59	25.78	8.50	9.38	9.94
74	E	3840	330	198	22.27	7.34	8.10	8.59	25.78	8.50	9.38	9.94
75	E	3840	330	198	22.27	7.34	8.10	8.59	25.78	8.50	9.38	9.94
76	E	3840	330	198	22.27	7.34	8.10	8.59	25.78	8.50	9.38	9.94
77	E	3840	330	198	22.27	7.34	8.10	8.59	25.78	8.50	9.38	9.94
78	E	3840	330	198	22.27	7.34	8.10	8.59	25.78	8.50	9.38	9.94
79	E	3440	242	104	24.85	6.01	6.77	7.26	28.78	6.96	7.84	8.41
80	E	3580	253	132	23.88	6.04	6.80	7.29	27.65	7.00	7.87	8.44
81	E	3640	286	143	23.49	6.72	7.47	7.96	27.20	7.78	8.66	9.22
82	E	3580	253	132	23.88	6.04	6.80	7.29	27.65	7.00	7.87	8.44
83	E	2950	220	70	28.98	6.38	7.13	7.62	33.56	7.38	8.26	8.82
84	E	2950	220	70	28.98	6.38	7.13	7.62	33.56	7.38	8.26	8.82
85	E	3290	270	80	25.99	7.02	7.77	8.26	30.09	8.12	9.00	9.57

86	E	3250	260	80	26.31	6.84	7.60	8.08	30.46	7.92	8.80	9.36
87	E	3230	230	70	26.47	6.09	6.85	7.33	30.65	7.05	7.93	8.49
88	E	3230	230	70	26.47	6.09	6.85	7.33	30.65	7.05	7.93	8.49
89	E	3860	270	160	22.15	5.98	6.74	7.23	25.65	6.92	7.80	8.37
90	E	3810	260	150	22.44	5.83	6.59	7.08	25.98	6.76	7.63	8.20
91	E	3860	280	160	22.15	6.20	6.96	7.45	25.65	7.18	8.06	8.62
92	E	3630	240	120	23.55	5.65	6.41	6.90	27.27	6.55	7.42	7.99
93	E	3730	260	140	22.92	5.96	6.72	7.20	26.54	6.90	7.78	8.34
94	E	4020	300	190	21.27	6.38	7.14	7.63	24.63	7.39	8.26	8.83
95	E	3250	260	80	26.31	6.84	7.60	8.08	30.46	7.92	8.80	9.36
96	E	3610	250	120	23.68	5.92	6.68	7.17	27.42	6.86	7.73	8.30
97	E	4070	310	200	21.01	6.51	7.27	7.76	24.32	7.54	8.42	8.98
98	E	3630	240	120	23.55	5.65	6.41	6.90	27.27	6.55	7.42	7.99
99	E	3660	240	120	23.36	5.61	6.36	6.85	27.05	6.49	7.37	7.93
100	E	3660	260	130	23.36	6.07	6.83	7.32	27.05	7.03	7.91	8.47
101	E	3660	240	120	23.36	5.61	6.36	6.85	27.05	6.49	7.37	7.93
102	E	3290	270	80	25.99	7.02	7.77	8.26	30.09	8.12	9.00	9.57
103	F	3990	270	165	21.43	5.79	6.54	7.03	24.81	6.70	7.58	8.14
104	F	3990	260	160	21.43	5.57	6.33	6.82	24.81	6.45	7.33	7.89
105	F	3900	250	150	21.92	5.48	6.24	6.73	25.38	6.35	7.22	7.79
106	F	3990	270	165	21.43	5.79	6.54	7.03	24.81	6.70	7.58	8.14
107	F	4030	320	180	21.22	6.79	7.55	8.03	24.57	7.86	8.74	9.30
108	F	3450	320	105	24.78	7.93	8.69	9.18	28.70	9.18	10.06	10.62
109	F	3960	260	160	21.59	5.61	6.37	6.86	25.00	6.50	7.38	7.94
110	F	3980	300	180	21.48	6.44	7.20	7.69	24.87	7.46	8.34	8.90
111	F	3740	260	130	22.86	5.94	6.70	7.19	26.47	6.88	7.76	8.32
112	F	3430	320	105	24.93	7.98	8.73	9.22	28.86	9.24	10.11	10.68
113	F	3060	280	80	27.94	7.82	8.58	9.07	32.35	9.06	9.94	10.50
114	F	3900	250	150	21.92	5.48	6.24	6.73	25.38	6.35	7.22	7.79
115	G	3111	210	80	27.48	5.77	6.53	7.02	31.82	6.68	7.56	8.12
116	G	3351	220	90	25.51	5.61	6.37	6.86	29.54	6.50	7.38	7.94
117	G	3219	210	90	26.56	5.58	6.33	6.82	30.75	6.46	7.34	7.90
118	G	3111	210	80	27.48	5.77	6.53	7.02	31.82	6.68	7.56	8.12
119	G	3478	250	120	24.58	6.15	6.90	7.39	28.46	7.12	7.99	8.56
120	G	3585	320	140	23.85	7.63	8.39	8.88	27.62	8.84	9.71	10.28
121	G	3585	290	100	23.85	6.92	7.67	8.16	27.62	8.01	8.88	9.45
122	G	3231	230	110	26.46	6.09	6.84	7.33	30.64	7.05	7.92	8.49
123	G	3219	210	90	26.56	5.58	6.33	6.82	30.75	6.46	7.34	7.90
124	G	3302	230	100	25.89	5.96	6.71	7.20	29.98	6.90	7.77	8.34
125	G	3478	270	120	24.58	6.64	7.39	7.88	28.46	7.69	8.56	9.13
126	G	3257	220	100	26.25	5.78	6.53	7.02	30.40	6.69	7.56	8.13
127	G	3290	290	100	25.99	7.54	8.29	8.78	30.09	8.73	9.60	10.17
128	G	3478	270	120	24.58	6.64	7.39	7.88	28.46	7.69	8.56	9.13
129	H	3600	220	100	23.75	5.23	5.98	6.47	27.50	6.05	6.93	7.49
130	H	3800	230	120	22.50	5.18	5.93	6.42	26.05	5.99	6.87	7.43
131	H	3800	230	120	22.50	5.18	5.93	6.42	26.05	5.99	6.87	7.43
132	H	3700	230	110	23.11	5.31	6.07	6.56	26.76	6.15	7.03	7.60

133	H	3800	230	120	22.50	5.18	5.93	6.42	26.05	5.99	6.87	7.43
134	H	3900	270	120	21.92	5.92	6.68	7.16	25.38	6.85	7.73	8.30
135	H	3800	230	120	22.50	5.18	5.93	6.42	26.05	5.99	6.87	7.43
136	H	3900	270	120	21.92	5.92	6.68	7.16	25.38	6.85	7.73	8.30
137	H	3800	230	120	22.50	5.18	5.93	6.42	26.05	5.99	6.87	7.43
138	H	3400	260	70	25.15	6.54	7.30	7.78	29.12	7.57	8.45	9.01
139	H	3800	230	120	22.50	5.18	5.93	6.42	26.05	5.99	6.87	7.43
140	H	3800	230	120	22.50	5.18	5.93	6.42	26.05	5.99	6.87	7.43
141	H	3800	230	120	22.50	5.18	5.93	6.42	26.05	5.99	6.87	7.43
142	H	3800	230	120	22.50	5.18	5.93	6.42	26.05	5.99	6.87	7.43
143	H	3800	230	120	22.50	5.18	5.93	6.42	26.05	5.99	6.87	7.43
144	H	3800	230	120	22.50	5.18	5.93	6.42	26.05	5.99	6.87	7.43
145	I	3403	210	90	25.12	5.28	6.03	6.52	29.09	6.11	6.99	7.55
146	I	3816	240	120	22.41	5.38	6.13	6.62	25.94	6.23	7.10	7.67
147	I	3831	280	120	22.32	6.25	7.01	7.49	25.84	7.24	8.11	8.68
148	I	3850	230	100	22.21	5.11	5.86	6.35	25.71	5.91	6.79	7.36
149	I	3803	260	120	22.48	5.85	6.60	7.09	26.03	6.77	7.64	8.21
150	I	3681	230	100	23.23	5.34	6.10	6.59	26.89	6.19	7.06	7.63
151	I	3842	230	120	22.25	5.12	5.88	6.36	25.77	5.93	6.80	7.37
152	I	3803	280	120	22.48	6.30	7.05	7.54	26.03	7.29	8.17	8.73
153	I	3817	240	120	22.40	5.38	6.13	6.62	25.94	6.22	7.10	7.67
154	I	3817	240	120	22.40	5.38	6.13	6.62	25.94	6.22	7.10	7.67
155	I	3831	280	120	22.32	6.25	7.01	7.49	25.84	7.24	8.11	8.68
156	I	3702	260	120	23.10	6.00	6.76	7.25	26.74	6.95	7.83	8.39
157	I	3500	250	120	24.43	6.11	6.86	7.35	28.29	7.07	7.95	8.51
158	I	3406	270	80	25.10	6.78	7.53	8.02	29.07	7.85	8.72	9.29
159	I	3406	270	80	25.10	6.78	7.53	8.02	29.07	7.85	8.72	9.29
160	I	3500	250	120	24.43	6.11	6.86	7.35	28.29	7.07	7.95	8.51
161	I	3800	260	120	22.50	5.85	6.61	7.09	26.05	6.77	7.65	8.21
162	I	3702	260	120	23.10	6.00	6.76	7.25	26.74	6.95	7.83	8.39
163	I	3939	260	120	21.71	5.64	6.40	6.89	25.13	6.53	7.41	7.98
164	I	3370	260	80	25.37	6.60	7.35	7.84	29.38	7.64	8.51	9.08
165	I	3946	260	120	21.67	5.63	6.39	6.88	25.09	6.52	7.40	7.96
166	I	3839	230	120	22.27	5.12	5.88	6.37	25.79	5.93	6.81	7.37
167	I	3370	260	80	25.37	6.60	7.35	7.84	29.38	7.64	8.51	9.08
168	J	3510	230	130	24.36	5.60	6.36	6.85	28.21	6.49	7.36	7.93
169	J	3564	260	140	23.99	6.24	6.99	7.48	27.78	7.22	8.10	8.66
170	J	3741	270	150	22.85	6.17	6.93	7.42	26.46	7.15	8.02	8.59
171	J	3737	260	150	22.88	5.95	6.71	7.19	26.49	6.89	7.76	8.33
172	J	3705	300	140	23.08	6.92	7.68	8.17	26.72	8.02	8.89	9.46
173	J	3510	230	130	24.36	5.60	6.36	6.85	28.21	6.49	7.36	7.93
174	J	3375	230	100	25.33	5.83	6.58	7.07	29.33	6.75	7.62	8.19
175	J	3934	300	190	21.73	6.52	7.28	7.76	25.17	7.55	8.43	8.99
176	J	3375	230	100	25.33	5.83	6.58	7.07	29.33	6.75	7.62	8.19
177	J	3734	250	150	22.90	5.72	6.48	6.97	26.51	6.63	7.50	8.07
178	J	3432	270	110	24.91	6.73	7.48	7.97	28.85	7.79	8.67	9.23
179	J	3510	230	130	24.36	5.60	6.36	6.85	28.21	6.49	7.36	7.93

180	J	3375	230	100	25.33	5.83	6.58	7.07	29.33	6.75	7.62	8.19
181	J	3839	290	170	22.27	6.46	7.22	7.70	25.79	7.48	8.36	8.92
182	J	3375	230	100	25.33	5.83	6.58	7.07	29.33	6.75	7.62	8.19
183	J	3432	270	110	24.91	6.73	7.48	7.97	28.85	7.79	8.67	9.23
184	J	3375	230	100	25.33	5.83	6.58	7.07	29.33	6.75	7.62	8.19
185	J	3432	270	110	24.91	6.73	7.48	7.97	28.85	7.79	8.67	9.23
186	K	3820	260	120	22.38	5.82	6.58	7.06	25.92	6.74	7.61	8.18
187	K	3750	240	100	22.80	5.47	6.23	6.72	26.40	6.34	7.21	7.78
188	K	3808	310	120	22.45	6.96	7.72	8.20	26.00	8.06	8.94	9.50
189	K	3808	310	120	22.45	6.96	7.72	8.20	26.00	8.06	8.94	9.50
190	K	3780	260	110	22.62	5.88	6.64	7.13	26.19	6.81	7.69	8.25
191	K	3916	320	140	21.83	6.99	7.74	8.23	25.28	8.09	8.97	9.53
192	K	4050	300	160	21.11	6.33	7.09	7.58	24.44	7.33	8.21	8.77
193	K	3820	260	120	22.38	5.82	6.58	7.06	25.92	6.74	7.61	8.18
194	K	3060	280	80	27.94	7.82	8.58	9.07	32.35	9.06	9.94	10.50
195	K	3808	300	120	22.45	6.74	7.49	7.98	26.00	7.80	8.68	9.24
196	K	3750	240	100	22.80	5.47	6.23	6.72	26.40	6.34	7.21	7.78
197	K	3750	260	110	22.80	5.93	6.69	7.17	26.40	6.86	7.74	8.31
198	K	3750	260	110	22.80	5.93	6.69	7.17	26.40	6.86	7.74	8.31
199	K	3200	230	100	26.72	6.15	6.90	7.39	30.94	7.12	7.99	8.56
200	K	3300	270	110	25.91	7.00	7.75	8.24	30.00	8.10	8.98	9.54
201	K	3750	260	110	22.80	5.93	6.69	7.17	26.40	6.86	7.74	8.31
202	L	4100	280	170	20.85	5.84	6.60	7.08	24.15	6.76	7.64	8.20
203	L	4300	250	150	19.88	4.97	5.73	6.22	23.02	5.76	6.63	7.20
204	L	4300	250	150	19.88	4.97	5.73	6.22	23.02	5.76	6.63	7.20
205	L	4100	280	170	20.85	5.84	6.60	7.08	24.15	6.76	7.64	8.20
206	L	4300	250	150	19.88	4.97	5.73	6.22	23.02	5.76	6.63	7.20
207	L	4000	240	110	21.38	5.13	5.89	6.37	24.75	5.94	6.82	7.38
208	L	4100	280	170	20.85	5.84	6.60	7.08	24.15	6.76	7.64	8.20
209	L	4000	240	110	21.38	5.13	5.89	6.37	24.75	5.94	6.82	7.38
210	L	4000	240	110	21.38	5.13	5.89	6.37	24.75	5.94	6.82	7.38
211	M	3925	270	140	21.78	5.88	6.64	7.13	25.22	6.81	7.69	8.25
212	M	3954	278	167	21.62	6.01	6.77	7.26	25.04	6.96	7.84	8.40
213	M	3872	289	156	22.08	6.38	7.14	7.63	25.57	7.39	8.27	8.83
214	M	3950	344	167	21.65	7.45	8.20	8.69	25.06	8.62	9.50	10.06
215	M	3921	289	189	21.81	6.30	7.06	7.55	25.25	7.30	8.17	8.74
216	N	3794	215	130	22.54	4.85	5.60	6.09	26.09	5.61	6.49	7.05
217	N	3705	215	130	23.08	4.96	5.72	6.21	26.72	5.74	6.62	7.19
218	N	3705	215	130	23.08	4.96	5.72	6.21	26.72	5.74	6.62	7.19
219	N	3702	215	130	23.10	4.97	5.72	6.21	26.74	5.75	6.63	7.19
220	N	3703	230	140	23.09	5.31	6.07	6.56	26.74	6.15	7.03	7.59
221	O	3700	280	120	23.11	6.47	7.23	7.71	26.76	7.49	8.37	8.93
222	O	3700	280	120	23.11	6.47	7.23	7.71	26.76	7.49	8.37	8.93
223	O	3600	240	100	23.75	5.70	6.46	6.94	27.50	6.60	7.48	8.04
224	O	3600	240	100	23.75	5.70	6.46	6.94	27.50	6.60	7.48	8.04
