# Supplementary material for: Assessment of the Nutritional Impact of the 10% Snack Recommendation in Pet Diets
Source: Vet Sci. 2025 Mar 18;12(3):282. doi: 10.3390/vetsci12030282 (PMC11945345; doi:10.3390/vetsci12030282)
Supplement: Supplementary file 1 [file vetsci-12-00282-s001.zip › Supplementary_Table2_CatsProtein.pdf]

Supplementary Table 2. Nutritional impact of 10% MER restriction with snack inclusion in protein intake of cats. according FEDIAF (2024)

					Protein									
					75 kcal/kg$^{0.67}$					100 kcal/kg$^{0.67}$				
					Minimum recommended protein 6.25 g per kg metabolic bodyweight									
Commercial dry diet	Brand	Metabolizable energy (kcal/kg)	Crude protein (g/kg)	Fat (g/kg)	Food consumption considering 90% of the maintenance energy requirement. per kg of metabolic weight	Amount of protein consumed per kg of metabolic weight	Protein consumed per kg of (BW)0.67 + protein from dry snacks	Protein consumed per kg of BW)0.67 + protein from churu	Protein consumed per kg of BW)0.67 + protein from wet snacks	Food consumption considering 90% of the maintenance energy requirement. per kg of metabolic weight	Amount of protein consumed per kg of metabolic weight	Protein consumed per kg of BW)0.67 + protein from dry snacks	Protein consumed per kg of BW)0.67 + protein from churu	Protein consumed per kg of BW)0.67 + protein from wet snacks
1	A	3919	310	120	17.22	5.34	6.13	6.86	6.38	22.97	7.12	8.18	9.15	8.51
2	A	3912	310	120	17.25	5.35	6.14	6.87	6.39	23.01	7.13	8.19	10.22	8.52
3	A	3800	365	105	17.76	6.48	7.28	8.00	7.53	23.68	8.64	9.70	11.73	10.03
4	A	3909	400	120	17.27	6.91	7.70	8.43	7.95	23.02	9.21	10.27	12.29	10.60
5	A	3800	365	100	17.76	6.48	7.28	8.00	7.53	23.68	8.64	9.70	11.73	10.03
6	A	4157	320	170	16.24	5.20	5.99	6.72	6.24	21.65	6.93	7.99	10.01	8.32
7	A	3990	310	140	16.92	5.24	6.04	6.77	6.29	22.56	6.99	8.05	10.08	8.38
8	A	3912	310	120	17.25	5.35	6.14	6.87	6.39	23.01	7.13	8.19	10.22	8.52
9	A	3919	340	120	17.22	5.86	6.65	7.38	6.90	22.97	7.81	8.87	10.89	9.20
10	A	4203	330	190	16.06	5.30	6.09	6.82	6.34	21.41	7.07	8.12	10.15	8.46
11	A	4082	320	180	16.54	5.29	6.08	6.81	6.33	22.05	7.06	8.11	10.14	8.44
12	A	4060	320	130	16.63	5.32	6.11	6.84	6.36	22.17	7.09	8.15	10.18	8.48
13	A	4010	330	140	16.83	5.55	6.35	7.08	6.60	22.44	7.41	8.46	10.49	8.80
14	A	4203	330	190	16.06	5.30	6.09	6.82	6.34	21.41	7.07	8.12	10.15	8.46
15	A	3909	400	120	17.27	6.91	7.70	8.43	7.95	23.02	9.21	10.27	12.29	10.60
16	A	4042	360	140	16.70	6.01	6.80	7.53	7.05	22.27	8.02	9.07	11.10	9.40
17	A	4197	340	180	16.08	5.47	6.26	6.99	6.51	21.44	7.29	8.35	10.38	8.68
18	A	4290	350	200	15.73	5.51	6.30	7.03	6.55	20.98	7.34	8.40	10.43	8.73
19	A	3912	310	120	17.25	5.35	6.14	6.87	6.39	23.01	7.13	8.19	10.22	8.52
20	A	4021	400	150	16.79	6.71	7.51	8.24	7.76	22.38	8.95	10.01	12.04	10.34
21	A	3813	410	90	17.70	7.26	8.05	8.78	8.30	23.60	9.68	10.73	12.76	11.07
22	A	4157	320	170	16.24	5.20	5.99	6.72	6.24	21.65	6.93	7.99	10.01	8.32
23	A	3840	350	190	17.58	6.15	6.95	7.67	7.19	23.44	8.20	9.26	11.29	9.59
24	A	4203	330	190	16.06	5.30	6.09	6.82	6.34	21.41	7.07	8.12	10.15	8.46
25	A	3840	365	105	17.58	6.42	7.21	7.94	7.46	23.44	8.55	9.61	11.64	9.94

26	A	4088	330	180	16.51	5.45	6.24	6.97	6.49	22.02	7.27	8.32	10.35	8.65
27	A	4290	350	200	15.73	5.51	6.30	7.03	6.55	20.98	7.34	8.40	10.43	8.73
28	A	4197	340	180	16.08	5.47	6.26	6.99	6.51	21.44	7.29	8.35	10.38	8.68
29	A	4082	320	180	16.54	5.29	6.08	6.81	6.33	22.05	7.06	8.11	10.14	8.44
30	A	3909	400	120	17.27	6.91	7.70	8.43	7.95	23.02	9.21	10.27	12.29	10.60
31	A	3909	400	120	17.27	6.91	7.70	8.43	7.95	23.02	9.21	10.27	12.29	10.60
32	A	4021	400	150	16.79	6.71	7.51	8.24	7.76	22.38	8.95	10.01	12.04	10.34
33	A	4263	350	190	15.83	5.54	6.33	7.06	6.58	21.11	7.39	8.45	10.47	8.78
34	A	4203	330	190	16.06	5.30	6.09	6.82	6.34	21.41	7.07	8.12	10.15	8.46
35	A	4203	330	190	16.06	5.30	6.09	6.82	6.34	21.41	7.07	8.12	10.15	8.46
36	A	4157	320	170	16.24	5.20	5.99	6.72	6.24	21.65	6.93	7.99	10.01	8.32
37	A	4157	320	170	16.24	5.20	5.99	6.72	6.24	21.65	6.93	7.99	10.01	8.32
38	A	3813	410	90	17.70	7.26	8.05	8.78	8.30	23.60	9.68	10.73	12.76	11.07
39	A	4143	250	200	16.29	4.07	4.87	5.59	5.11	21.72	5.43	6.49	8.52	6.82
40	B	3820	360	140	17.67	6.36	7.15	7.88	7.40	23.56	8.48	9.54	11.57	9.87
41	B	3480	360	100	19.40	6.98	7.78	8.50	8.02	25.86	9.31	10.37	12.40	10.70
42	B	3480	360	100	19.40	6.98	7.78	8.50	8.02	25.86	9.31	10.37	12.40	10.70
43	B	3660	320	110	18.44	5.90	6.69	7.42	6.94	24.59	7.87	8.93	10.95	9.26
44	B	3660	320	110	18.44	5.90	6.69	7.42	6.94	24.59	7.87	8.93	10.95	9.26
45	B	3480	360	100	19.40	6.98	7.78	8.50	8.02	25.86	9.31	10.37	12.40	10.70
46	B	3750	460	110	18.00	8.28	9.07	9.80	9.32	24.00	11.04	12.10	14.13	12.43
47	B	4420	330	215	15.27	5.04	5.83	6.56	6.08	20.36	6.72	7.78	9.80	8.11
48	B	3380	280	90	19.97	5.59	6.38	7.11	6.63	26.63	7.46	8.51	10.54	8.84
49	B	3400	400	90	19.85	7.94	8.73	9.46	8.98	26.47	10.59	11.65	13.67	11.98
50	B	4200	330	165	16.07	5.30	6.10	6.82	6.35	21.43	7.07	8.13	10.16	8.46
51	B	3660	320	110	18.44	5.90	6.69	7.42	6.94	24.59	7.87	8.93	10.95	9.26
52	B	4160	300	160	16.23	4.87	5.66	6.39	5.91	21.63	6.49	7.55	9.58	7.88
53	B	4370	420	200	15.45	6.49	7.28	8.01	7.53	20.59	8.65	9.71	11.74	10.04
54	B	4370	420	200	15.45	6.49	7.28	8.01	7.53	20.59	8.65	9.71	11.74	10.04
55	B	4380	440	200	15.41	6.78	7.57	8.30	7.82	20.55	9.04	10.10	12.13	10.43
56	B	4390	440	200	15.38	6.77	7.56	8.29	7.81	20.50	9.02	10.08	12.11	10.41
57	B	4056	360	200	16.64	5.99	6.78	7.51	7.03	22.19	7.99	9.05	11.07	9.38
58	B	4056	360	200	16.64	5.99	6.78	7.51	7.03	22.19	7.99	9.05	11.07	9.38
59	B	3447	380	100	19.58	7.44	8.23	8.96	8.48	26.11	9.92	10.98	13.01	11.31

60	B	4097	380	220	16.48	6.26	7.05	7.78	7.30	21.97	8.35	9.40	11.43	9.74
61	B	4105	420	200	16.44	6.91	7.70	8.43	7.95	21.92	9.21	10.27	12.29	10.60
62	B	4132	440	200	16.34	7.19	7.98	8.71	8.23	21.78	9.58	10.64	12.67	10.97
63	B	3569	460	110	18.91	8.70	9.49	10.22	9.74	25.22	11.60	12.66	14.69	12.99
64	B	4223	440	220	15.98	7.03	7.83	8.55	8.07	21.31	9.38	10.43	12.46	10.77
65	B	4987.00	80.00	440.00	18.00	85.00	13.54	5.96	6.75	7.48	7.00	18.05	7.94	9.00
66	B	4800.00	80.00	440.00	18.00	85.00	14.06	6.19	6.98	7.71	7.23	18.75	8.25	9.31
67	B	4800.00	80.00	420.00	18.00	89.00	14.06	5.91	6.70	7.43	6.95	18.75	7.88	8.93
68	B	4800.00	90.00	440.00	22.00	85.00	14.06	6.19	6.98	7.71	7.23	18.75	8.25	9.31
69	B	4170.00	80.00	460.00	51.00	89.00	16.19	7.45	8.24	8.97	8.49	21.58	9.93	10.99
70	C	3632.00	80.00	350.00	70.00	92.00	18.58	6.50	7.30	8.03	7.55	24.78	8.67	9.73
71	C	4278.00	80.00	310.00	25.00	77.00	15.78	4.89	5.68	6.41	5.93	21.04	6.52	7.58
72	C	3972.00	80.00	310.00	38.00	79.00	16.99	5.27	6.06	6.79	6.31	22.66	7.02	8.08
73	C	3815.00	80.00	280.00	70.00	63.00	17.69	4.95	5.75	6.48	6.00	23.59	6.61	7.66
74	C	3457.00	80.00	380.00	80.00	83.00	19.53	7.42	8.21	8.94	8.46	26.03	9.89	10.95
75	C	3867.00	80.00	300.00	50.00	79.00	17.46	5.24	6.03	6.76	6.28	23.27	6.98	8.04
76	C	4200.00	80.00	280.00	57.00	75.00	16.07	4.50	5.29	6.02	5.54	21.43	6.00	7.06
77	C	3771.00	80.00	265.00	53.00	85.00	17.90	4.74	5.54	6.26	5.78	23.87	6.32	7.38
78	C	3459.00	80.00	320.00	107.00	90.00	19.51	6.24	7.04	7.77	7.29	26.02	8.33	9.38
79	C	4435.00	80.00	320.00	26.00	83.00	15.22	4.87	5.66	6.39	5.91	20.29	6.49	7.55
80	C	3439.00	80.00	320.00	116.00	84.00	19.63	6.28	7.07	7.80	7.32	26.17	8.37	9.43
81	C	3730.00	80.00	320.00	86.00	92.00	18.10	5.79	6.58	7.31	6.83	24.13	7.72	8.78
82	C	4136.00	80.00	310.00	62.00	76.00	16.32	5.06	5.85	6.58	6.10	21.76	6.75	7.80
83	C	3921.00	80.00	290.00	64.00	86.00	17.21	4.99	5.79	6.51	6.03	22.95	6.66	7.71
84	C	3760.00	80.00	250.00	53.00	72.00	17.95	4.49	5.28	6.01	5.53	23.94	5.98	7.04
85	C	3632.00	80.00	350.00	70.00	92.00	18.58	6.50	7.30	8.03	7.55	24.78	8.67	9.73
86	C	3815.00	80.00	280.00	70.00	63.00	17.69	4.95	5.75	6.48	6.00	23.59	6.61	7.66
87	C	3867.00	80.00	300.00	50.00	79.00	17.46	5.24	6.03	6.76	6.28	23.27	6.98	8.04
88	C	3771.00	80.00	265.00	85.00	53.00	17.90	4.74	5.54	6.26	5.78	23.87	6.32	7.38
89	C	4278.00	80.00	310.00	25.00	77.00	15.78	4.89	5.68	6.41	5.93	21.04	6.52	7.58
90	C	4136.00	80.00	310.00	62.00	76.00	16.32	5.06	5.85	6.58	6.10	21.76	6.75	7.80
91	C	3730.00	80.00	320.00	86.00	92.00	18.10	5.79	6.58	7.31	6.83	24.13	7.72	8.78
92	C	3970.00	80.00	360.00	29.00	88.00	17.00	6.12	6.91	7.64	7.16	22.67	8.16	9.22
93	D	3890.00	100.00	310.00	40.00	95.00	17.35	5.38	6.17	6.90	6.42	23.14	7.17	8.23

94	D	3850.00	100.00	360.00	45.00	95.00	17.53	6.31	7.10	7.83	7.35	23.38	8.42	9.47
95	D	3850.00	100.00	360.00	45.00	95.00	17.53	6.31	7.10	7.83	7.35	23.38	8.42	9.47
96	D	3890.00	100.00	310.00	40.00	95.00	17.35	5.38	6.17	6.90	6.42	23.14	7.17	8.23
97	D	3850.00	100.00	360.00	45.00	95.00	17.53	6.31	7.10	7.83	7.35	23.38	8.42	9.47
98	D	3850.00	100.00	360.00	45.00	95.00	17.53	6.31	7.10	7.83	7.35	23.38	8.42	9.47
99	D	3890.00	100.00	310.00	40.00	95.00	17.35	5.38	6.17	6.90	6.42	23.14	7.17	8.23
100	E	4200.00	120.00	360.00	25.00	75.00	16.07	5.79	6.58	7.31	6.83	21.43	7.71	8.77
101	E	3950.00	120.00	400.00	40.00	85.00	17.09	6.84	7.63	8.36	7.88	22.78	9.11	10.17
102	F	3600.00	100.00	360.00	40.00	82.00	18.75	6.75	7.54	8.27	7.79	25.00	9.00	10.06
103	F	3600.00	100.00	360.00	40.00	82.00	18.75	6.75	7.54	8.27	7.79	25.00	9.00	10.06
104	F	3600.00	100.00	360.00	40.00	82.00	18.75	6.75	7.54	8.27	7.79	25.00	9.00	10.06
105	F	3600.00	100.00	360.00	40.00	82.00	18.75	6.75	7.54	8.27	7.79	25.00	9.00	10.06
106	F	3950.00	100.00	320.00	30.00	80.00	17.09	5.47	6.26	6.99	6.51	22.78	7.29	8.35
107	F	3950.00	100.00	320.00	30.00	80.00	17.09	5.47	6.26	6.99	6.51	22.78	7.29	8.35
108	F	3950.00	100.00	320.00	30.00	80.00	17.09	5.47	6.26	6.99	6.51	22.78	7.29	8.35
109	F	4000.00	100.00	350.00	30.00	82.00	16.88	5.91	6.70	7.43	6.95	22.50	7.88	8.93
110	F	3600.00	100.00	360.00	40.00	82.00	18.75	6.75	7.54	8.27	7.79	25.00	9.00	10.06
111	F	3600.00	100.00	360.00	40.00	82.00	18.75	6.75	7.54	8.27	7.79	25.00	9.00	10.06
112	F	3600.00	100.00	360.00	40.00	82.00	18.75	6.75	7.54	8.27	7.79	25.00	9.00	10.06
113	G	3800.00	100.00	360.00	45.00	70.00	17.76	6.39	7.19	7.92	7.44	23.68	8.53	9.58
114	G	3800.00	100.00	360.00	45.00	70.00	17.76	6.39	7.19	7.92	7.44	23.68	8.53	9.58
115	G	3800.00	100.00	360.00	45.00	70.00	17.76	6.39	7.19	7.92	7.44	23.68	8.53	9.58
116	G	4042.00	100.00	360.00	45.00	70.00	16.70	6.01	6.80	7.53	7.05	22.27	8.02	9.07
117	G	3912.00	100.00	310.00	35.00	80.00	17.25	5.35	6.14	6.87	6.39	23.01	7.13	8.19
118	G	3912.00	100.00	310.00	35.00	80.00	17.25	5.35	6.14	6.87	6.39	23.01	7.13	8.19
119	G	3912.00	100.00	310.00	35.00	80.00	17.25	5.35	6.14	6.87	6.39	23.01	7.13	8.19
120	G	4060.00	100.00	320.00	35.00	80.00	16.63	5.32	6.11	6.84	6.36	22.17	7.09	8.15
121	H	4232.00	80.00	310.00	40.00	90.00	15.95	4.94	5.74	6.47	5.99	21.27	6.59	7.65
122	H	3206.00	110.00	290.00	100.00	100.00	21.05	6.11	6.90	7.63	7.15	28.07	8.14	9.20
123	I	4180.00	90.00	440.00	25.00	75.00	16.15	7.11	7.90	8.63	8.15	21.53	9.47	10.53
124	I	3810.00	90.00	460.00	60.00	75.00	17.72	8.15	8.94	9.67	9.19	23.62	10.87	11.92
