# Supplementary material for: Assessment of the Nutritional Impact of the 10% Snack Recommendation in Pet Diets
Source: Vet Sci. 2025 Mar 18;12(3):282. doi: 10.3390/vetsci12030282 (PMC11945345; doi:10.3390/vetsci12030282)
Supplement: Supplementary file 1 [file vetsci-12-00282-s001.zip › Supplementary_Table3_DogFat.pdf]

Supplementary Table 3. Nutritional impact of 10% MER restriction with snack inclusion in fat intake of dogs. according FEDIAF (2024)

					Fat							
					95 kcal/kg$^{0.75}$				110 kcal/kg$^{0.75}$			
					Minimum recommended fat 1.51 g per kg metabolic bodyweight							
**Commercial dry diet**	**Brand**	**Metabolizable energy**	**Crude protein (g/kg)**	**Fat (g/kg)**	**Food consumption considering 90% of the maintenance energy requirement. per kg of metabolic weight**	**Amount of fat consumed per kg of metabolic weight**	**Fat consumed per kg of (BW)0.75 + fat from dry snacks**	**Fat consumed per kg of (BW)0.75 + fat from wet snacks**	**Food consumption considering 90% of the maintenance energy requirement. per kg of metabolic weight**	**Amount of fat consumed per kg of metabolic weight**	**Fat consumed per kg of (BW)0.75 + fat from dry snacks**	**Fat consumed per kg of (BW)0.75 + fat from wet snacks**
1	A	3869	280	160	22.10	3.54	3.77	4.06	25.59	4.09	4.36	4.70
2	A	4058	280	180	21.07	3.79	4.03	4.32	24.40	4.39	4.66	5.00
3	A	3901	280	140	21.92	3.07	3.30	3.60	25.38	3.55	3.82	4.16
4	A	3667	270	140	23.32	3.26	3.50	3.79	27.00	3.78	4.05	4.39
5	A	3888	270	160	21.99	3.52	3.75	4.05	25.46	4.07	4.34	4.68
6	A	3783	290	140	22.60	3.16	3.40	3.69	26.17	3.66	3.93	4.27
7	A	3899	260	160	21.93	3.51	3.74	4.04	25.39	4.06	4.33	4.67
8	A	3993	270	180	21.41	3.85	4.09	4.38	24.79	4.46	4.73	5.07
9	A	4124	290	180	20.73	3.73	3.96	4.26	24.01	4.32	4.59	4.93
10	A	4142	290	180	20.64	3.72	3.95	4.24	23.90	4.30	4.57	4.91
11	A	4142	300	180	20.64	3.72	3.95	4.24	23.90	4.30	4.57	4.91
12	A	3598	230	110	23.76	2.61	2.85	3.14	27.52	3.03	3.30	3.64
13	A	3667	270	140	23.32	3.26	3.50	3.79	27.00	3.78	4.05	4.39
14	A	3695	280	110	23.14	2.55	2.78	3.07	26.79	2.95	3.22	3.56
15	A	3830	310	120	22.32	2.68	2.91	3.21	25.85	3.10	3.37	3.71
16	A	3961	220	180	21.59	3.89	4.12	4.41	24.99	4.50	4.77	5.11
17	A	3964	260	160	21.57	3.45	3.68	3.98	24.97	4.00	4.27	4.61
18	A	4095	240	180	20.88	3.76	3.99	4.29	24.18	4.35	4.62	4.96
19	A	3691	220	120	23.16	2.78	3.01	3.31	26.82	3.22	3.49	3.83
20	A	3859	260	120	22.16	2.66	2.89	3.19	25.65	3.08	3.35	3.69
21	A	4092	240	160	20.89	3.34	3.58	3.87	24.19	3.87	4.14	4.48
22	A	3953	220	170	21.63	3.68	3.91	4.20	25.04	4.26	4.53	4.87

23	A	4156	220	160	20.57	3.29	3.52	3.82	23.82	3.81	4.08	4.42
24	A	4055	240	180	21.09	3.80	4.03	4.32	24.41	4.39	4.66	5.00
25	A	4003	240	150	21.36	3.20	3.44	3.73	24.73	3.71	3.98	4.32
26	A	3920	220	150	21.81	3.27	3.50	3.80	25.26	3.79	4.06	4.40
27	A	4009	250	140	21.33	2.99	3.22	3.51	24.69	3.46	3.73	4.07
28	A	3839	230	120	22.27	2.67	2.91	3.20	25.79	3.09	3.36	3.70
29	A	3963	240	150	21.57	3.24	3.47	3.76	24.98	3.75	4.02	4.36
30	A	4161	260	180	20.55	3.70	3.93	4.23	23.79	4.28	4.55	4.89
31	A	3118	320	80	27.42	2.19	2.43	2.72	31.75	2.54	2.81	3.15
32	A	3144	250	90	27.19	2.45	2.68	2.97	31.49	2.83	3.10	3.44
33	A	3263	280	90	26.20	2.36	2.59	2.89	30.34	2.73	3.00	3.34
34	A	3165	250	90	27.01	2.43	2.66	2.96	31.28	2.82	3.08	3.43
35	B	3800	280	130	22.50	2.93	3.16	3.45	26.05	3.39	3.66	4.00
36	B	3980	260	150	21.48	3.22	3.46	3.75	24.87	3.73	4.00	4.34
37	B	3740	280	120	22.86	2.74	2.98	3.27	26.47	3.18	3.45	3.79
38	B	3810	240	105	22.44	2.36	2.59	2.88	25.98	2.73	3.00	3.34
39	C	4097	290	180	20.87	3.76	3.99	4.28	24.16	4.35	4.62	4.96
40	C	4123	290	180	20.74	3.73	3.97	4.26	24.01	4.32	4.59	4.93
41	C	3931	290	140	21.75	3.05	3.28	3.57	25.18	3.53	3.80	4.14
42	C	4213	290	190	20.29	3.86	4.09	4.38	23.50	4.46	4.73	5.08
43	C	4020	280	160	21.27	3.40	3.64	3.93	24.63	3.94	4.21	4.55
44	C	4040	260	160	21.16	3.39	3.62	3.91	24.50	3.92	4.19	4.53
45	C	4040	260	160	21.16	3.39	3.62	3.91	24.50	3.92	4.19	4.53
46	C	3920	260	140	21.81	3.05	3.29	3.58	25.26	3.54	3.81	4.15
47	C	3555	280	80	24.05	1.92	2.16	2.45	27.85	2.23	2.50	2.84
48	C	3555	280	80	24.05	1.92	2.16	2.45	27.85	2.23	2.50	2.84
49	C	3601	315	115	23.74	2.73	2.96	3.26	27.49	3.16	3.43	3.77
50	C	4015	250	160	21.30	3.41	3.64	3.93	24.66	3.95	4.21	4.56
51	C	3724	260	140	22.96	3.21	3.45	3.74	26.58	3.72	3.99	4.33
52	C	2979	355	80	28.70	2.30	2.53	2.82	33.23	2.66	2.93	3.27
53	C	2979	355	80	28.70	2.30	2.53	2.82	33.23	2.66	2.93	3.27

54	C	3550	340	140	24.08	3.37	3.60	3.90	27.89	3.90	4.17	4.51
55	C	3550	340	140	24.08	3.37	3.60	3.90	27.89	3.90	4.17	4.51
56	C	3831	200	130	22.32	2.90	3.13	3.43	25.84	3.36	3.63	3.97
57	C	4000	240	180	21.38	3.85	4.08	4.37	24.75	4.46	4.72	5.07
58	D	3875	311	211	22.06	4.66	4.89	5.18	25.55	5.39	5.66	6.00
59	D	4250	300	180	20.12	3.62	3.85	4.15	23.29	4.19	4.46	4.80
60	D	4250	300	180	20.12	3.62	3.85	4.15	23.29	4.19	4.46	4.80
61	D	4150	280	170	20.60	3.50	3.74	4.03	23.86	4.06	4.32	4.67
62	D	4150	280	170	20.60	3.50	3.74	4.03	23.86	4.06	4.32	4.67
63	D	4150	280	170	20.60	3.50	3.74	4.03	23.86	4.06	4.32	4.67
64	D	4050	280	160	21.11	3.38	3.61	3.90	24.44	3.91	4.18	4.52
65	D	4050	280	160	21.11	3.38	3.61	3.90	24.44	3.91	4.18	4.52
66	D	4050	280	160	21.11	3.38	3.61	3.90	24.44	3.91	4.18	4.52
67	D	3610	264	165	23.68	3.90	4.14	4.43	27.42	4.52	4.79	5.13
68	D	3480	286	132	24.57	3.24	3.47	3.77	28.45	3.75	4.02	4.36
69	D	3680	275	165	23.23	3.83	4.06	4.36	26.90	4.43	4.70	5.04
70	D	3870	308	187	22.09	4.13	4.36	4.65	25.58	4.78	5.05	5.39
71	E	3660	240	120	23.36	2.80	3.04	3.33	27.05	3.25	3.52	3.86
72	E	3660	240	120	23.36	2.80	3.04	3.33	27.05	3.25	3.52	3.86
73	E	3840	330	198	22.27	4.40	4.64	4.93	25.78	5.10	5.37	5.71
74	E	3840	330	198	22.27	4.40	4.64	4.93	25.78	5.10	5.37	5.71
75	E	3840	330	198	22.27	4.40	4.64	4.93	25.78	5.10	5.37	5.71
76	E	3840	330	198	22.27	4.40	4.64	4.93	25.78	5.10	5.37	5.71
77	E	3840	330	198	22.27	4.40	4.64	4.93	25.78	5.10	5.37	5.71
78	E	3840	330	198	22.27	4.40	4.64	4.93	25.78	5.10	5.37	5.71
79	E	3440	242	104	24.85	2.59	2.83	3.12	28.78	3.00	3.27	3.61
80	E	3580	253	132	23.88	3.15	3.38	3.68	27.65	3.65	3.92	4.26
81	E	3640	286	143	23.49	3.36	3.59	3.88	27.20	3.89	4.16	4.50
82	E	3580	253	132	23.88	3.15	3.38	3.68	27.65	3.65	3.92	4.26
83	E	2950	220	70	28.98	2.03	2.26	2.56	33.56	2.35	2.62	2.96
84	E	2950	220	70	28.98	2.03	2.26	2.56	33.56	2.35	2.62	2.96

85	E	3290	270	80	25.99	2.08	2.31	2.61	30.09	2.41	2.68	3.02
86	E	3250	260	80	26.31	2.10	2.34	2.63	30.46	2.44	2.71	3.05
87	E	3230	230	70	26.47	1.85	2.09	2.38	30.65	2.15	2.42	2.76
88	E	3230	230	70	26.47	1.85	2.09	2.38	30.65	2.15	2.42	2.76
89	E	3860	270	160	22.15	3.54	3.78	4.07	25.65	4.10	4.37	4.71
90	E	3810	260	150	22.44	3.37	3.60	3.89	25.98	3.90	4.17	4.51
91	E	3860	280	160	22.15	3.54	3.78	4.07	25.65	4.10	4.37	4.71
92	E	3630	240	120	23.55	2.83	3.06	3.35	27.27	3.27	3.54	3.88
93	E	3730	260	140	22.92	3.21	3.44	3.74	26.54	3.72	3.99	4.33
94	E	4020	300	190	21.27	4.04	4.27	4.57	24.63	4.68	4.95	5.29
95	E	3250	260	80	26.31	2.10	2.34	2.63	30.46	2.44	2.71	3.05
96	E	3610	250	120	23.68	2.84	3.07	3.37	27.42	3.29	3.56	3.90
97	E	4070	310	200	21.01	4.20	4.43	4.73	24.32	4.86	5.13	5.48
98	E	3630	240	120	23.55	2.83	3.06	3.35	27.27	3.27	3.54	3.88
99	E	3660	240	120	23.36	2.80	3.04	3.33	27.05	3.25	3.52	3.86
100	E	3660	260	130	23.36	3.04	3.27	3.56	27.05	3.52	3.79	4.13
101	E	3660	240	120	23.36	2.80	3.04	3.33	27.05	3.25	3.52	3.86
102	E	3290	270	80	25.99	2.08	2.31	2.61	30.09	2.41	2.68	3.02
103	F	3990	270	165	21.43	3.54	3.77	4.06	24.81	4.09	4.36	4.70
104	F	3990	260	160	21.43	3.43	3.66	3.96	24.81	3.97	4.24	4.58
105	F	3900	250	150	21.92	3.29	3.52	3.82	25.38	3.81	4.08	4.42
106	F	3990	270	165	21.43	3.54	3.77	4.06	24.81	4.09	4.36	4.70
107	F	4030	320	180	21.22	3.82	4.05	4.35	24.57	4.42	4.69	5.03
108	F	3450	320	105	24.78	2.60	2.83	3.13	28.70	3.01	3.28	3.62
109	F	3960	260	160	21.59	3.45	3.69	3.98	25.00	4.00	4.27	4.61
110	F	3980	300	180	21.48	3.87	4.10	4.39	24.87	4.48	4.75	5.09
111	F	3740	260	130	22.86	2.97	3.20	3.50	26.47	3.44	3.71	4.05
112	F	3430	320	105	24.93	2.62	2.85	3.14	28.86	3.03	3.30	3.64
113	F	3060	280	80	27.94	2.24	2.47	2.76	32.35	2.59	2.86	3.20
114	F	3900	250	150	21.92	3.29	3.52	3.82	25.38	3.81	4.08	4.42
115	G	3111	210	80	27.48	2.20	2.43	2.73	31.82	2.55	2.82	3.16

116	G	3351	220	90	25.51	2.30	2.53	2.82	29.54	2.66	2.93	3.27
117	G	3219	210	90	26.56	2.39	2.62	2.92	30.75	2.77	3.04	3.38
118	G	3111	210	80	27.48	2.20	2.43	2.73	31.82	2.55	2.82	3.16
119	G	3478	250	120	24.58	2.95	3.18	3.48	28.46	3.42	3.69	4.03
120	G	3585	320	140	23.85	3.34	3.57	3.87	27.62	3.87	4.14	4.48
121	G	3585	290	100	23.85	2.38	2.62	2.91	27.62	2.76	3.03	3.37
122	G	3231	230	110	26.46	2.91	3.14	3.44	30.64	3.37	3.64	3.98
123	G	3219	210	90	26.56	2.39	2.62	2.92	30.75	2.77	3.04	3.38
124	G	3302	230	100	25.89	2.59	2.82	3.12	29.98	3.00	3.27	3.61
125	G	3478	270	120	24.58	2.95	3.18	3.48	28.46	3.42	3.69	4.03
126	G	3257	220	100	26.25	2.63	2.86	3.15	30.40	3.04	3.31	3.65
127	G	3290	290	100	25.99	2.60	2.83	3.13	30.09	3.01	3.28	3.62
128	G	3478	270	120	24.58	2.95	3.18	3.48	28.46	3.42	3.69	4.03
129	H	3600	220	100	23.75	2.38	2.61	2.90	27.50	2.75	3.02	3.36
130	H	3800	230	120	22.50	2.70	2.93	3.23	26.05	3.13	3.40	3.74
131	H	3800	230	120	22.50	2.70	2.93	3.23	26.05	3.13	3.40	3.74
132	H	3700	230	110	23.11	2.54	2.77	3.07	26.76	2.94	3.21	3.55
133	H	3800	230	120	22.50	2.70	2.93	3.23	26.05	3.13	3.40	3.74
134	H	3900	270	120	21.92	2.63	2.86	3.16	25.38	3.05	3.32	3.66
135	H	3800	230	120	22.50	2.70	2.93	3.23	26.05	3.13	3.40	3.74
136	H	3900	270	120	21.92	2.63	2.86	3.16	25.38	3.05	3.32	3.66
137	H	3800	230	120	22.50	2.70	2.93	3.23	26.05	3.13	3.40	3.74
138	H	3400	260	70	25.15	1.76	1.99	2.29	29.12	2.04	2.31	2.65
139	H	3800	230	120	22.50	2.70	2.93	3.23	26.05	3.13	3.40	3.74
140	H	3800	230	120	22.50	2.70	2.93	3.23	26.05	3.13	3.40	3.74
141	H	3800	230	120	22.50	2.70	2.93	3.23	26.05	3.13	3.40	3.74
142	H	3800	230	120	22.50	2.70	2.93	3.23	26.05	3.13	3.40	3.74
143	H	3800	230	120	22.50	2.70	2.93	3.23	26.05	3.13	3.40	3.74
144	H	3800	230	120	22.50	2.70	2.93	3.23	26.05	3.13	3.40	3.74
145	I	3403	210	90	25.12	2.26	2.49	2.79	29.09	2.62	2.89	3.23
146	I	3816	240	120	22.41	2.69	2.92	3.22	25.94	3.11	3.38	3.72

147	I	3831	280	120	22.32	2.68	2.91	3.21	25.84	3.10	3.37	3.71
148	I	3850	230	100	22.21	2.22	2.45	2.75	25.71	2.57	2.84	3.18
149	I	3803	260	120	22.48	2.70	2.93	3.22	26.03	3.12	3.39	3.73
150	I	3681	230	100	23.23	2.32	2.56	2.85	26.89	2.69	2.96	3.30
151	I	3842	230	120	22.25	2.67	2.90	3.20	25.77	3.09	3.36	3.70
152	I	3803	280	120	22.48	2.70	2.93	3.22	26.03	3.12	3.39	3.73
153	I	3817	240	120	22.40	2.69	2.92	3.22	25.94	3.11	3.38	3.72
154	I	3817	240	120	22.40	2.69	2.92	3.22	25.94	3.11	3.38	3.72
155	I	3831	280	120	22.32	2.68	2.91	3.21	25.84	3.10	3.37	3.71
156	I	3702	260	120	23.10	2.77	3.00	3.30	26.74	3.21	3.48	3.82
157	I	3500	250	120	24.43	2.93	3.16	3.46	28.29	3.39	3.66	4.00
158	I	3406	270	80	25.10	2.01	2.24	2.54	29.07	2.33	2.59	2.94
159	I	3406	270	80	25.10	2.01	2.24	2.54	29.07	2.33	2.59	2.94
160	I	3500	250	120	24.43	2.93	3.16	3.46	28.29	3.39	3.66	4.00
161	I	3800	260	120	22.50	2.70	2.93	3.23	26.05	3.13	3.40	3.74
162	I	3702	260	120	23.10	2.77	3.00	3.30	26.74	3.21	3.48	3.82
163	I	3939	260	120	21.71	2.60	2.84	3.13	25.13	3.02	3.29	3.63
164	I	3370	260	80	25.37	2.03	2.26	2.56	29.38	2.35	2.62	2.96
165	I	3946	260	120	21.67	2.60	2.83	3.13	25.09	3.01	3.28	3.62
166	I	3839	230	120	22.27	2.67	2.91	3.20	25.79	3.09	3.36	3.70
167	I	3370	260	80	25.37	2.03	2.26	2.56	29.38	2.35	2.62	2.96
168	J	3510	230	130	24.36	3.17	3.40	3.69	28.21	3.67	3.94	4.28
169	J	3564	260	140	23.99	3.36	3.59	3.89	27.78	3.89	4.16	4.50
170	J	3741	270	150	22.85	3.43	3.66	3.96	26.46	3.97	4.24	4.58
171	J	3737	260	150	22.88	3.43	3.66	3.96	26.49	3.97	4.24	4.58
172	J	3705	300	140	23.08	3.23	3.46	3.76	26.72	3.74	4.01	4.35
173	J	3510	230	130	24.36	3.17	3.40	3.69	28.21	3.67	3.94	4.28
174	J	3375	230	100	25.33	2.53	2.77	3.06	29.33	2.93	3.20	3.54
175	J	3934	300	190	21.73	4.13	4.36	4.66	25.17	4.78	5.05	5.39
176	J	3375	230	100	25.33	2.53	2.77	3.06	29.33	2.93	3.20	3.54
177	J	3734	250	150	22.90	3.43	3.67	3.96	26.51	3.98	4.25	4.59

178	J	3432	270	110	24.91	2.74	2.97	3.27	28.85	3.17	3.44	3.78
179	J	3510	230	130	24.36	3.17	3.40	3.69	28.21	3.67	3.94	4.28
180	J	3375	230	100	25.33	2.53	2.77	3.06	29.33	2.93	3.20	3.54
181	J	3839	290	170	22.27	3.79	4.02	4.31	25.79	4.38	4.65	4.99
182	J	3375	230	100	25.33	2.53	2.77	3.06	29.33	2.93	3.20	3.54
183	J	3432	270	110	24.91	2.74	2.97	3.27	28.85	3.17	3.44	3.78
184	J	3375	230	100	25.33	2.53	2.77	3.06	29.33	2.93	3.20	3.54
185	J	3432	270	110	24.91	2.74	2.97	3.27	28.85	3.17	3.44	3.78
186	K	3820	260	120	22.38	2.69	2.92	3.21	25.92	3.11	3.38	3.72
187	K	3750	240	100	22.80	2.28	2.51	2.81	26.40	2.64	2.91	3.25
188	K	3808	310	120	22.45	2.69	2.93	3.22	26.00	3.12	3.39	3.73
189	K	3808	310	120	22.45	2.69	2.93	3.22	26.00	3.12	3.39	3.73
190	K	3780	260	110	22.62	2.49	2.72	3.02	26.19	2.88	3.15	3.49
191	K	3916	320	140	21.83	3.06	3.29	3.58	25.28	3.54	3.81	4.15
192	K	4050	300	160	21.11	3.38	3.61	3.90	24.44	3.91	4.18	4.52
193	K	3820	260	120	22.38	2.69	2.92	3.21	25.92	3.11	3.38	3.72
194	K	3060	280	80	27.94	2.24	2.47	2.76	32.35	2.59	2.86	3.20
195	K	3808	300	120	22.45	2.69	2.93	3.22	26.00	3.12	3.39	3.73
196	K	3750	240	100	22.80	2.28	2.51	2.81	26.40	2.64	2.91	3.25
197	K	3750	260	110	22.80	2.51	2.74	3.04	26.40	2.90	3.17	3.51
198	K	3750	260	110	22.80	2.51	2.74	3.04	26.40	2.90	3.17	3.51
199	K	3200	230	100	26.72	2.67	2.90	3.20	30.94	3.09	3.36	3.70
200	K	3300	270	110	25.91	2.85	3.08	3.38	30.00	3.30	3.57	3.91
201	K	3750	260	110	22.80	2.51	2.74	3.04	26.40	2.90	3.17	3.51
202	L	4100	280	170	20.85	3.55	3.78	4.07	24.15	4.10	4.37	4.72
203	L	4300	250	150	19.88	2.98	3.22	3.51	23.02	3.45	3.72	4.06
204	L	4300	250	150	19.88	2.98	3.22	3.51	23.02	3.45	3.72	4.06
205	L	4100	280	170	20.85	3.55	3.78	4.07	24.15	4.10	4.37	4.72
206	L	4300	250	150	19.88	2.98	3.22	3.51	23.02	3.45	3.72	4.06
207	L	4000	240	110	21.38	2.35	2.58	2.88	24.75	2.72	2.99	3.33
208	L	4100	280	170	20.85	3.55	3.78	4.07	24.15	4.10	4.37	4.72

209	L	4000	240	110	21.38	2.35	2.58	2.88	24.75	2.72	2.99	3.33
210	L	4000	240	110	21.38	2.35	2.58	2.88	24.75	2.72	2.99	3.33
211	M	3925	270	140	21.78	3.05	3.28	3.58	25.22	3.53	3.80	4.14
212	M	3954	278	167	21.62	3.61	3.84	4.14	25.04	4.18	4.45	4.79
213	M	3872	289	156	22.08	3.44	3.68	3.97	25.57	3.99	4.26	4.60
214	M	3950	344	167	21.65	3.61	3.85	4.14	25.06	4.19	4.46	4.80
215	M	3921	289	189	21.81	4.12	4.35	4.65	25.25	4.77	5.04	5.38
216	N	3794	215	130	22.54	2.93	3.16	3.46	26.09	3.39	3.66	4.00
217	N	3705	215	130	23.08	3.00	3.23	3.53	26.72	3.47	3.74	4.08
218	N	3705	215	130	23.08	3.00	3.23	3.53	26.72	3.47	3.74	4.08
219	N	3702	215	130	23.10	3.00	3.24	3.53	26.74	3.48	3.75	4.09
220	N	3703	230	140	23.09	3.23	3.47	3.76	26.74	3.74	4.01	4.35
221	O	3700	280	120	23.11	2.77	3.01	3.30	26.76	3.21	3.48	3.82
222	O	3700	280	120	23.11	2.77	3.01	3.30	26.76	3.21	3.48	3.82
223	O	3600	240	100	23.75	2.38	2.61	2.90	27.50	2.75	3.02	3.36
224	O	3600	240	100	23.75	2.38	2.61	2.90	27.50	2.75	3.02	3.36
