# Supplementary material for: Assessment of the Nutritional Impact of the 10% Snack Recommendation in Pet Diets
Source: Vet Sci. 2025 Mar 18;12(3):282. doi: 10.3390/vetsci12030282 (PMC11945345; doi:10.3390/vetsci12030282)
Supplement: Supplementary file 1 [file vetsci-12-00282-s001.zip › Supplementary_Table4_CatFat.pdf]

Supplementary Table 4. Nutritional impact of 10% MER restriction with snack inclusion in fat intake of cats. according FEDIAF (2024)

					Fat									
					75 kcal/kg$^{0.67}$					100 kcal/kg$^{0.67}$				
					Minimum recommended fat 2.25 g per kg metabolic bodyweight									
**Commercial dry diet**	**Brand**	**Metabolizable energy (kcal/kg)**	**Crude Protein (g/kg)**	**Fat (g/kg)**	**Food consumption considering 90% of the maintenance energy requirement. per kg of metabolic weight**	**Amount of fat consumed per kg of metabolic weight**	**Fat consumed per kg of (BW)0.67 + fat from dry snacks**	**Fat consumed per kg of (BW)0.67 + fat from churu**	**Fat consumed per kg of (BW)0.67 + fat from wet snacks**	**Food consumption considering 90% of the maintenance energy requirement. per kg of metabolic weight**	**Amount of fat consumed per kg of metabolic weight**	**Fat consumed per kg of (BW)0.67 + fat from dry snacks**	**Fat consumed per kg of (BW)0.67 + fat from churu**	**Fat consumed per kg of (BW)0.67 + fat from wet snacks**
1	A	3919	310	120	17.22	2.07	2.30	2.21	2.32	23.71	2.84	3.15	3.03	3.19
2	A	3912	310	120	17.25	2.07	2.30	2.21	2.33	23.71	2.84	3.15	3.03	3.19
3	A	3800	365	105	17.76	1.87	2.10	2.00	2.12	23.83	2.50	2.81	2.69	2.84
4	A	3909	400	120	17.27	2.07	2.30	2.21	2.33	23.34	2.80	3.11	2.99	3.14
5	A	3800	365	100	17.76	1.78	2.01	1.91	2.03	23.97	2.40	2.71	2.58	2.74
6	A	4157	320	170	16.24	2.76	2.99	2.90	3.02	22.22	3.78	4.09	3.96	4.12
7	A	3990	310	140	16.92	2.37	2.60	2.51	2.62	23.16	3.24	3.55	3.43	3.58
8	A	3912	310	120	17.25	2.07	2.30	2.21	2.33	23.71	2.84	3.15	3.03	3.19
9	A	3919	340	120	17.22	2.07	2.30	2.21	2.32	23.46	2.81	3.12	3.00	3.16
10	A	4203	330	190	16.06	3.05	3.28	3.19	3.31	21.92	4.16	4.47	4.35	4.51
11	A	4082	320	180	16.54	2.98	3.21	3.11	3.23	22.07	3.97	4.28	4.16	4.31
12	A	4060	320	130	16.63	2.16	2.39	2.30	2.42	23.35	3.04	3.35	3.22	3.38
13	A	4010	330	140	16.83	2.36	2.59	2.50	2.61	23.00	3.22	3.53	3.40	3.56
14	A	4203	330	190	16.06	3.05	3.28	3.19	3.31	21.92	4.16	4.47	4.35	4.51
15	A	3909	400	120	17.27	2.07	2.30	2.21	2.33	23.34	2.80	3.11	2.99	3.14
16	A	4042	360	140	16.70	2.34	2.57	2.48	2.59	22.83	3.20	3.51	3.38	3.54
17	A	4197	340	180	16.08	2.89	3.13	3.03	3.15	21.92	3.95	4.26	4.13	4.29
18	A	4290	350	200	15.73	3.15	3.38	3.29	3.40	21.63	4.33	4.64	4.51	4.67
19	A	3912	310	120	17.25	2.07	2.30	2.21	2.33	23.71	2.84	3.15	3.03	3.19
20	A	4021	400	150	16.79	2.52	2.75	2.66	2.77	22.57	3.38	3.69	3.57	3.73
21	A	3813	410	90	17.70	1.59	1.83	1.73	1.85	24.59	2.21	2.52	2.40	2.55

22	A	4157	320	170	16.24	2.76	2.99	2.90	3.02	22.22	3.78	4.09	3.96	4.12
23	A	3840	350	190	17.58	3.34	3.57	3.48	3.60	21.56	4.10	4.41	4.28	4.44
24	A	4203	330	190	16.06	3.05	3.28	3.19	3.31	21.92	4.16	4.47	4.35	4.51
25	A	3840	365	105	17.58	1.85	2.08	1.98	2.10	23.83	2.50	2.81	2.69	2.84
26	A	4088	330	180	16.51	2.97	3.20	3.11	3.23	21.99	3.96	4.27	4.14	4.30
27	A	4290	350	200	15.73	3.15	3.38	3.29	3.40	21.63	4.33	4.64	4.51	4.67
28	A	4197	340	180	16.08	2.89	3.13	3.03	3.15	21.92	3.95	4.26	4.13	4.29
29	A	4082	320	180	16.54	2.98	3.21	3.11	3.23	22.07	3.97	4.28	4.16	4.31
30	A	3909	400	120	17.27	2.07	2.30	2.21	2.33	23.34	2.80	3.11	2.99	3.14
31	A	3909	400	120	17.27	2.07	2.30	2.21	2.33	23.34	2.80	3.11	2.99	3.14
32	A	4021	400	150	16.79	2.52	2.75	2.66	2.77	22.57	3.38	3.69	3.57	3.73
33	A	4263	350	190	15.83	3.01	3.24	3.15	3.26	21.56	4.10	4.41	4.28	4.44
34	A	4203	330	190	16.06	3.05	3.28	3.19	3.31	21.92	4.16	4.47	4.35	4.51
35	A	4203	330	190	16.06	3.05	3.28	3.19	3.31	21.92	4.16	4.47	4.35	4.51
36	A	4157	320	170	16.24	2.76	2.99	2.90	3.02	22.22	3.78	4.09	3.96	4.12
37	A	4157	320	170	16.24	2.76	2.99	2.90	3.02	22.22	3.78	4.09	3.96	4.12
38	A	3813	410	90	17.70	1.59	1.83	1.73	1.85	24.59	2.21	2.52	2.40	2.55
39	A	4143	250	200	16.29	3.26	3.49	3.40	3.51	21.72	4.34	4.65	4.53	4.69
40	B	3820	360	140	17.67	2.47	2.71	2.61	2.73	21.98	3.08	3.39	3.26	3.42
41	B	3480	360	100	19.40	1.94	2.17	2.08	2.20	23.94	2.39	2.70	2.58	2.73
42	B	3480	360	100	19.40	1.94	2.17	2.08	2.20	23.94	2.39	2.70	2.58	2.73
43	B	3660	320	110	18.44	2.03	2.26	2.17	2.28	23.06	2.54	2.85	2.72	2.88
44	B	3660	320	110	18.44	2.03	2.26	2.17	2.28	23.06	2.54	2.85	2.72	2.88
45	B	3480	360	100	19.40	1.94	2.17	2.08	2.20	23.94	2.39	2.70	2.58	2.73
46	B	3750	460	110	18.00	1.98	2.21	2.12	2.24	23.22	2.55	2.86	2.74	2.90
47	B	4420	330	215	15.27	3.28	3.52	3.42	3.54	20.61	4.43	4.74	4.62	4.77
48	B	3380	280	90	19.97	1.80	2.03	1.94	2.05	25.06	2.26	2.57	2.44	2.60
49	B	3400	400	90	19.85	1.79	2.02	1.93	2.04	25.43	2.29	2.60	2.47	2.63
50	B	4200	330	165	16.07	2.65	2.88	2.79	2.91	21.79	3.60	3.91	3.78	3.94
51	B	3660	320	110	18.44	2.03	2.26	2.17	2.28	23.06	2.54	2.85	2.72	2.88
52	B	4160	300	160	16.23	2.60	2.83	2.73	2.85	22.23	3.56	3.87	3.74	3.90
53	B	4370	420	200	15.45	3.09	3.32	3.23	3.35	20.52	4.10	4.41	4.29	4.44

54	B	4370	420	200	15.45	3.09	3.32	3.23	3.35	20.52	4.10	4.41	4.29	4.44
55	B	4380	440	200	15.41	3.08	3.31	3.22	3.34	20.39	4.08	4.39	4.26	4.42
56	B	4390	440	200	15.38	3.08	3.31	3.21	3.33	20.39	4.08	4.39	4.26	4.42
57	B	4056	360	200	16.64	3.33	3.56	3.47	3.58	20.77	4.15	4.46	4.34	4.50
58	B	4056	360	200	16.64	3.33	3.56	3.47	3.58	20.77	4.15	4.46	4.34	4.50
59	B	3447	380	100	19.58	1.96	2.19	2.10	2.21	24.07	2.41	2.72	2.59	2.75
60	B	4097	380	220	16.48	3.62	3.86	3.76	3.88	20.50	4.51	4.82	4.69	4.85
61	B	4105	420	200	16.44	3.29	3.52	3.43	3.54	20.32	4.06	4.37	4.25	4.41
62	B	4132	440	200	16.34	3.27	3.50	3.41	3.52	20.13	4.03	4.34	4.21	4.37
63	B	3569	460	110	18.91	2.08	2.31	2.22	2.34	22.94	2.52	2.83	2.71	2.86
64	B	4223	440	220	15.98	3.52	3.75	3.65	3.77	19.73	4.34	4.65	4.52	4.68
65	B	4987	440	220	13.54	2.98	3.21	3.12	3.23	19.73	4.34	4.65	4.52	4.68
66	B	4800	440	200	14.06	2.81	3.04	2.95	3.07	20.13	4.03	4.34	4.21	4.37
67	B	4800	420	200	14.06	2.81	3.04	2.95	3.07	20.32	4.06	4.37	4.25	4.41
68	B	4800	440	200	14.06	2.81	3.04	2.95	3.07	20.39	4.08	4.39	4.26	4.42
69	B	4170	460	110	16.19	1.78	2.01	1.92	2.04	22.94	2.52	2.83	2.71	2.86
70	C	3632	350	100	18.58	1.86	2.09	2.00	2.11	24.71	2.47	2.78	2.66	2.81
71	C	4278	310	200	15.78	3.16	3.39	3.29	3.41	21.00	4.20	4.51	4.38	4.54
72	C	3972	310	140	16.99	2.38	2.61	2.52	2.64	22.79	3.19	3.50	3.38	3.53
73	C	3815	280	130	17.69	2.30	2.53	2.44	2.56	23.83	3.10	3.41	3.28	3.44
74	C	3457	380	80	19.53	1.56	1.79	1.70	1.82	25.13	2.01	2.32	2.19	2.35
75	C	3867	300	130	17.46	2.27	2.50	2.41	2.53	23.45	3.05	3.36	3.23	3.39
76	C	4200	280	200	16.07	3.21	3.45	3.35	3.47	21.95	4.39	4.70	4.58	4.73
77	C	3771	265	120	17.90	2.15	2.38	2.29	2.40	24.23	2.91	3.22	3.09	3.25
78	C	3459	320	100	19.51	1.95	2.18	2.09	2.21	26.07	2.61	2.92	2.79	2.95
79	C	4435	320	230	15.22	3.50	3.73	3.64	3.76	20.41	4.69	5.00	4.88	5.04
80	C	3439	320	100	19.63	1.96	2.20	2.10	2.22	26.22	2.62	2.93	2.81	2.96
81	C	3730	320	130	18.10	2.35	2.58	2.49	2.61	24.58	3.20	3.51	3.38	3.54
82	C	4136	310	200	16.32	3.26	3.50	3.40	3.52	21.89	4.38	4.69	4.56	4.72
83	C	3921	290	180	17.21	3.10	3.33	3.24	3.35	22.75	4.10	4.40	4.28	4.44
84	C	3760	250	110	17.95	1.97	2.21	2.11	2.23	24.36	2.68	2.99	2.86	3.02
85	C	3632	350	100	18.58	1.86	2.09	2.00	2.11	24.71	2.47	2.78	2.66	2.81

86	C	3815	280	130	17.69	2.30	2.53	2.44	2.56	23.83	3.10	3.41	3.28	3.44
87	C	3867	300	130	17.46	2.27	2.50	2.41	2.53	23.45	3.05	3.36	3.23	3.39
88	C	3771	265	120	17.90	2.15	2.38	2.29	2.40	24.45	2.93	3.24	3.12	3.28
89	C	4278	310	200	15.78	3.16	3.39	3.29	3.41	21.00	4.20	4.51	4.38	4.54
90	C	4136	310	200	16.32	3.26	3.50	3.40	3.52	21.89	4.38	4.69	4.56	4.72
91	C	3730	320	130	18.10	2.35	2.58	2.49	2.61	24.58	3.20	3.51	3.38	3.54
92	C	3970	360	130	17.00	2.21	2.44	2.35	2.47	22.61	2.94	3.25	3.12	3.28
93	D	3890	310	120	17.35	2.08	2.31	2.22	2.34	24.18	2.90	3.21	3.09	3.24
94	D	3850	360	100	17.53	1.75	1.99	1.89	2.01	24.46	2.45	2.76	2.63	2.79
95	D	3850	360	100	17.53	1.75	1.99	1.89	2.01	24.46	2.45	2.76	2.63	2.79
96	D	3890	310	120	17.35	2.08	2.31	2.22	2.34	24.18	2.90	3.21	3.09	3.24
97	D	3850	360	100	17.53	1.75	1.99	1.89	2.01	24.46	2.45	2.76	2.63	2.79
98	D	3850	360	100	17.53	1.75	1.99	1.89	2.01	24.46	2.45	2.76	2.63	2.79
99	D	3890	310	120	17.35	2.08	2.31	2.22	2.34	24.18	2.90	3.21	3.09	3.24
100	E	4200	360	150	16.07	2.41	2.64	2.55	2.67	22.56	3.38	3.69	3.57	3.73
101	E	3950	400	110	17.09	1.88	2.11	2.02	2.14	23.93	2.63	2.94	2.82	2.97
102	F	3600	360	90	18.75	1.69	1.92	1.83	1.94	24.32	2.19	2.50	2.37	2.53
103	F	3600	360	90	18.75	1.69	1.92	1.83	1.94	24.32	2.19	2.50	2.37	2.53
104	F	3600	360	90	18.75	1.69	1.92	1.83	1.94	24.32	2.19	2.50	2.37	2.53
105	F	3600	360	90	18.75	1.69	1.92	1.83	1.94	24.32	2.19	2.50	2.37	2.53
106	F	3950	320	120	17.09	2.05	2.28	2.19	2.31	23.49	2.82	3.13	3.00	3.16
107	F	3950	320	120	17.09	2.05	2.28	2.19	2.31	23.49	2.82	3.13	3.00	3.16
108	F	3950	320	120	17.09	2.05	2.28	2.19	2.31	23.49	2.82	3.13	3.00	3.16
109	F	4000	350	120	16.88	2.03	2.26	2.16	2.28	23.28	2.79	3.10	2.98	3.14
110	F	3600	360	90	18.75	1.69	1.92	1.83	1.94	24.32	2.19	2.50	2.37	2.53
111	F	3600	360	90	18.75	1.69	1.92	1.83	1.94	24.32	2.19	2.50	2.37	2.53
112	F	3600	360	90	18.75	1.69	1.92	1.83	1.94	24.32	2.19	2.50	2.37	2.53
113	G	3800	360	100	17.76	1.78	2.01	1.91	2.03	23.90	2.39	2.70	2.58	2.73
114	G	3800	360	100	17.76	1.78	2.01	1.91	2.03	23.90	2.39	2.70	2.58	2.73
115	G	3800	360	100	17.76	1.78	2.01	1.91	2.03	23.90	2.39	2.70	2.58	2.73
116	G	4042	360	140	16.70	2.34	2.57	2.48	2.59	22.83	3.20	3.51	3.38	3.54
117	G	3912	310	120	17.25	2.07	2.30	2.21	2.33	23.71	2.84	3.15	3.03	3.19

118	G	3912	310	120	17.25	2.07	2.30	2.21	2.33	23.71	2.84	3.15	3.03	3.19
119	G	3912	310	120	17.25	2.07	2.30	2.21	2.33	23.71	2.84	3.15	3.03	3.19
120	G	4060	320	130	16.63	2.16	2.39	2.30	2.42	23.35	3.04	3.35	3.22	3.38
121	H	4232	310	210	15.95	3.35	3.58	3.49	3.61	21.36	4.49	4.80	4.67	4.83
122	H	3206	290	70	21.05	1.47	1.71	1.61	1.73	28.32	1.98	2.29	2.17	2.32
123	I	4180	440	200	16.15	3.23	3.46	3.37	3.49	20.30	4.06	4.37	4.24	4.40
124	I	3810	460	120	17.72	2.13	2.36	2.26	2.38	22.86	2.74	3.05	2.93	3.08
